# Metabolic switch from fatty acid oxidation to glycolysis in knock‐in mouse model of Barth syndrome

**DOI:** 10.15252/emmm.202317399

**Published:** 2023-08-03

**Authors:** Arpita Chowdhury, Angela Boshnakovska, Abhishek Aich, Aditi Methi, Ana Maria Vergel Leon, Ivan Silbern, Christian Lüchtenborg, Lukas Cyganek, Jan Prochazka, Radislav Sedlacek, Jiri Lindovsky, Dominic Wachs, Zuzana Nichtova, Dagmar Zudova, Gizela Koubkova, André Fischer, Henning Urlaub, Britta Brügger, Dörthe M Katschinski, Jan Dudek, Peter Rehling

**Affiliations:** ^1^ Department of Cellular Biochemistry University Medical Center Göttingen Göttingen Germany; ^2^ Cluster of Excellence “Multiscale Bioimaging: from Molecular Machines to Networks of Excitable Cells” (MBExC) University of Göttingen Göttingen Germany; ^3^ Department of Psychiatry and Psychotherapy University Medical Center Göttingen Göttingen Germany; ^4^ Department for Epigenetics and Systems Medicine in Neurodegenerative Diseases German Center for Neurodegenerative Diseases (DZNE) Göttingen Germany; ^5^ Department of Cardiovascular Physiology University Medical Center Göttingen Göttingen Germany; ^6^ The Bioanalytical Mass Spectrometry Group Max Planck Institute for Multidisciplinary Sciences Göttingen Germany; ^7^ Institute for Clinical Chemistry, University Medical Center Göttingen Göttingen Germany; ^8^ Heidelberg University Biochemistry Center (BZH) Heidelberg Germany; ^9^ DZHK (German Center for Cardiovascular Research) partner site Göttingen Göttingen Germany; ^10^ Stem Cell Unit, Clinic for Cardiology and Pneumology University Medical Center Göttingen, Georg‐August University Göttingen Göttingen Germany; ^11^ Czech Centre for Phenogenomics Institute of Molecular Genetics of the CAS Prague Czech Republic; ^12^ Max Planck Institute for Multidisciplinary Science Göttingen Germany; ^13^ Present address: Dewpoint Therapeutics GmbH Dresden Germany

**Keywords:** Barth syndrome, cardiolipin, cardiomyopathy, mitochondria, tafazzin, Genetics, Gene Therapy & Genetic Disease, Organelles

## Abstract

Mitochondria are central for cellular metabolism and energy supply. Barth syndrome (BTHS) is a severe disorder, due to dysfunction of the mitochondrial cardiolipin acyl transferase tafazzin. Altered cardiolipin remodeling affects mitochondrial inner membrane organization and function of membrane proteins such as transporters and the oxidative phosphorylation (OXPHOS) system. Here, we describe a mouse model that carries a G197V exchange in tafazzin, corresponding to BTHS patients. TAZ^G197V^ mice recapitulate disease‐specific pathology including cardiac dysfunction and reduced oxidative phosphorylation. We show that mutant mitochondria display defective fatty acid‐driven oxidative phosphorylation due to reduced levels of carnitine palmitoyl transferases. A metabolic switch in ATP production from OXPHOS to glycolysis is apparent in mouse heart and patient iPSC cell‐derived cardiomyocytes. An increase in glycolytic ATP production inactivates AMPK causing altered metabolic signaling in TAZ^G197V^. Treatment of mutant cells with AMPK activator reestablishes fatty acid‐driven OXPHOS and protects mice against cardiac dysfunction.

## Graphical abstract

Defective cardiolipin remodeling due to mutation in tafazzin causes Barth syndrome (BTHS) with cardiac impairment and reduced oxidative phosphorylation. Dysfunctional FAO‐driven OXPHOS due to altered AMPK signaling is restored in BTHS models by treatment with AMPK activator.

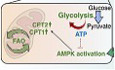

**The paper explained**

**Problem**

Cardiomyopathy and heart failure are hallmarks and a major cause for death in Barth syndrome (BTHS) patients. BTHS is caused by mutations in Tafazzin, a mitochondrial acyltransferase required for remodeling of the mitochondrial phospholipid cardiolipin. Why an alteration in mitochondrial lipid composition leads to cardiomyopathy is still not understood, thus resulting in poor targeted therapies against BTHS.

**Results**

We established a new mouse model for Barth syndrome that recapitulates a mutation found in patients. An elevated glycolytic metabolism is apparent in the mouse heart and in iPS cell‐derived cardiomyocytes of a corresponding Barth syndrome patient. Increased glycolytic ATP production inactivates AMPK signaling, resulting in defective mitochondrial fatty acid utilization leading to cardiac malfunction. Pharmacologic administration of AMPK activator reverses cellular metabolism *in vitro* and *in vivo*, protecting the heart against functional decline.

**Impact**

Our results provide new insights in the pathology of Barth syndrome and the mechanism underlying metabolic reprogramming linked to heart failure. Pharmacologic activation of AMPK, improving the cardiac outcome in mice, creates new avenue for therapeutic development in BTHS patients.

## Introduction

Barth syndrome (BTHS) is a X‐linked genetic disorder of lipid metabolism, which is caused by mutations in a nuclear gene encoding the mitochondrial acyltransferase Tafazzin (TAZ; Dudek & Maack, 2017). It is a rare and fatal mitochondrial disease, estimated to occur approximately 1 in every 400,000 individuals globally, exclusively affecting males. The common clinical symptoms of BTHS patients are cardiomyopathy, skeletal myopathy, neutropenia, growth retardation, organic aciduria, and recurrent infections (Clarke *et al*, 2013). The cardiomyopathy phenotype is the major clinical feature diagnosed in 70% of patients within their first year of life and includes dilated and hypertrophic cardiomyopathy and left ventricular non‐compaction (Pang *et al*, 2022). In these patients along with neutropenia, cardiomyopathy is known to be the major cause of lethality. There are no specific treatments or cure for BTHS known till date. The current treatment of BTHS cardiomyopathy mainly aims at symptom reduction (Finsterer, 2019). This could well be attributed to the poor knowledge of the underlying mechanism leading to cardiomyopathy due to malfunctioning of Tafazzin.

Tafazzin, which is associated with the mitochondrial membranes is a monolysocardiolipin (MLCL) transacylase that transfers unsaturated fatty acyl chains from phosphatidylcholine to MLCL to produce mature cardiolipin (CL). Cardiolipin, which is a signature phospholipid of mitochondria, helps to maintain the mitochondrial ultrastructure and contributes to the activity of the oxidative phosphorylation system since it is required for proper activity of the mitochondrial electron transport chain (ETC) and the ATP synthase. Accordingly, loss of tafazzin and the concomitant altered cardiolipin composition primarily affect enzymes of the inner mitochondrial membrane, leading to reduced ETC activity and Ca^2+^ transport by MCU, increased production of reactive oxygen species (ROS), and decreased ATP synthesis. As the heart heavily depends on constant ATP production, it is severely affected and thus cardiac dysfunction is a major cause for lethality in BTHS patients. However, as loss of tafazzin has pleiotropic effects on mitochondrial physiology, the mechanisms that lead to cardiomyopathy are still obscure.

To study BTHS, several experimental models have been reported previously (Acehan *et al*, 2011; Ren *et al*, 2019; Wang *et al*, 2020; Zhu *et al*, 2021) including doxycycline‐induced short hairpin RNA Taz knockdown (KD) mice (Acehan *et al*, 2011; Ren *et al*, 2019), *Taz* global knockout (gKO) mice (Wang *et al*, 2020), *Taz* cardiomyocyte‐specific knockout (cKO) mice (Wang *et al*, 2020; Zhu *et al*, 2021), as well as mouse embryonic stem cell (ESC)‐derived cardiomyocytes (Acehan *et al*, 2009), and human‐induced pluripotent stem cell (iPSC)‐derived cardiomyocytes (Wang *et al*, 2014; Dudek *et al*, 2016). Yet, all of these models come with certain limitations, and currently, there is no animal model available that mimics a BTHS causing mutation.

Here, we generated a knock‐in patient mutation *TAZ* mouse. We selected a missense mutation c.590 G > T in the exon 7 in the conserved structural core of the mouse *TAZ* gene, resulting in a G197V substitution at a highly conserved residue. Previously we showed that patient derived cardiomyocytes carrying the same mutation displayed severe functional defects (Dudek *et al*, 2016). We show that the TAZ^G197V^ mouse model displays the typical pathology found in iPSC cell‐derived cardiomyocytes with significant deterioration of cardiac function over time. Previously defined biochemical and molecular abnormalities in the cardiac mitochondria are present in this mouse model. We find that a metabolic imbalance between fatty acid metabolism and glycolysis in BTHS mouse heart tissue and in patient‐derived iPSC‐cardiomyocytes contributes to the heart failure process. We report on a decrease in fatty acid metabolism due to aberrant AMPK signaling. An increase in glycolysis in TAZ^G197V^ maintains higher amounts of ATP in mutant cardiomyocytes rendering AMPK inactive. Pharmacological AMPK activation reversed the metabolic defect to normal levels of fatty acid metabolism. Therefore, establishing a patient mutation mouse model enabled us to dissect a metabolic reorganization in BTHS heart models that contributes to cardiac dysfunction.

## Results

### Generation of a patient mutation knock‐in Barth syndrome mouse model

**Figure 1:**
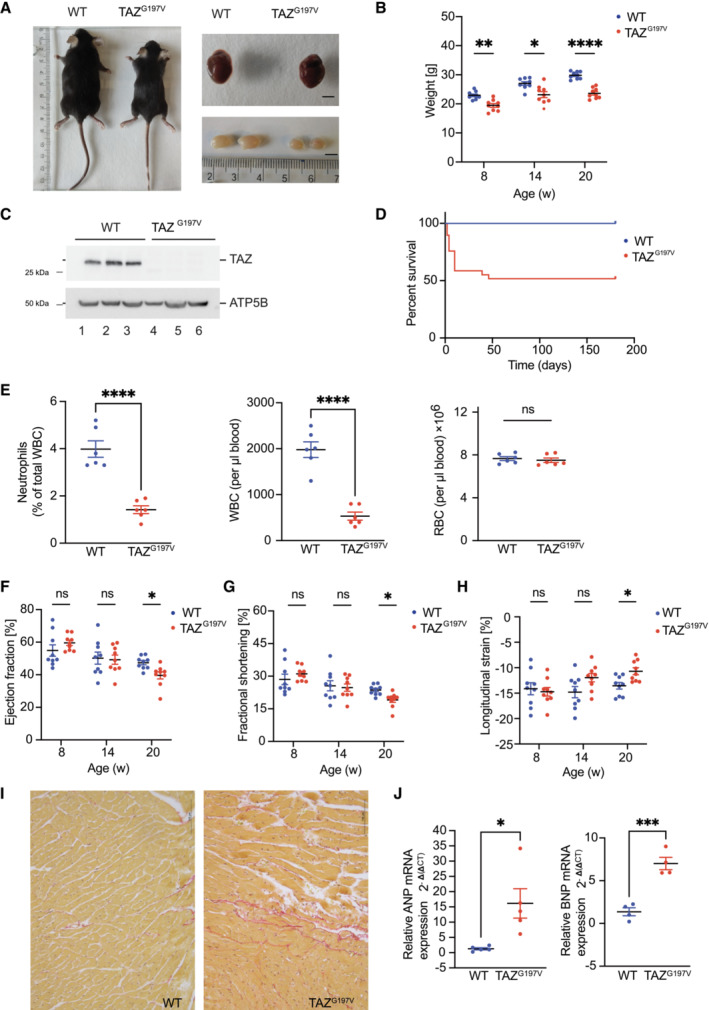
Characterization of the TAZ ^G197V^ mouse model ARepresentative pictures of mouse body size, dissected heart, and testis from 12 weeks old wild type (WT) and TAZ^G197V^ mice. Bar, 0.5 cm.BBody weight of animals at indicated age. Mean ± SEM, *n* = 9, 2Way ANOVA: *P < 0.05, **P < 0.01, ****P < 0.0001.CWestern blot analysis of isolated mouse heart mitochondria from 12‐week‐old animals.DSurvival of indicated mice presented as percent of total number of mice analyzed. *n* = 60 (per genotype), median survival of TAZ^G197V^ mice is 24.5 weeks, Mantel‐Cox test: ****P < 0.0001.EQuantification of neutrophils, percent of white blood cells (left), white blood cells (middle), and red blood cells (right) of 12‐week‐old mice. Mean ± SEM, *n* = 6, unpaired *t*‐test: ****P < 0.0001, ns (non‐significant).F–HEchocardiography of wild type and TAZ^G197V^ mice. (F) Ejection fraction from short axis expressed in %, (G) Fractional shortening in %, and (H) Longitudinal systolic strain expressed in %. Mean ± SEM, *n* = 9, 2‐way ANOVA: *P < 0.05.IPicrosirius red staining of histology sections of hearts from WT and TAZ mutant mice.JqPCR analysis of ANP (left) and BNP (right) from total mRNA isolated from heart lysates of 20‐week‐old mice. Mean ± SEM, *n* = 5 for ANP, *n* = 4 for BNP, unpaired *t*‐test: *P < 0.05, ***P < 0.001. Source data are available online for this figure.

**Figure EV1:**
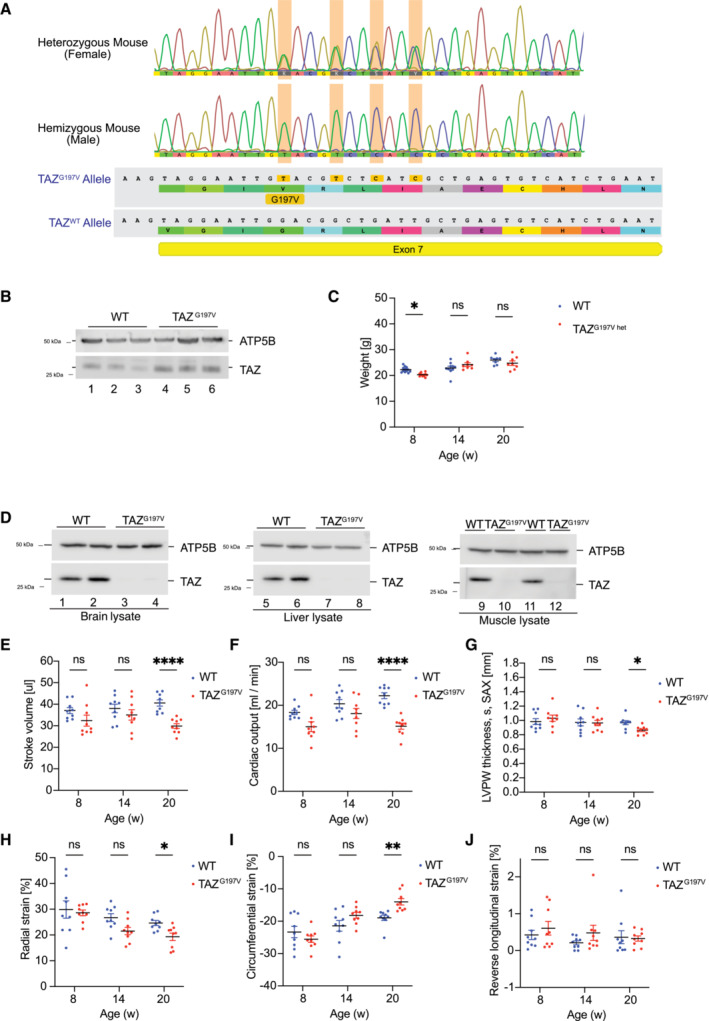
Characterization of the TAZ^G197V^ mouse model APresentation of Crispr/Cas9 generated mutation in Exon 7 in the gene encoding Tafazzin.BWestern blot analysis of steady state protein levels from isolated heart mitochondria of 20‐week‐old female TAZ^G197V^ heterozygous mice.CBody weight of female WT and TAZ^G197V^ heterozygous mice. Mean ± SEM, *n* = 8, 2Way ANOVA Multiple comparisons: *P < 0.05.DSteady state protein levels analyzed by western blotting of isolated mitochondria from brain, liver, and muscle of 12‐week‐old male mice.E–JQuantification of stroke volume (E), cardiac output from parasternal short axis (SAX) (F), left ventricle posterior systolic wall thickness analyzed from parasternal short axis (SAX) (G), Radial systolic strain (H) Circumferential systolic strain (I), And longitudinal diastolic strain (J). Mean ± SEM, *n* = 9, 2Way ANOVA Multiple comparisons: *P < 0.05, **P < 0.01, ****P < 0.0001.

To define defects in mitochondrial function and their pathophysiological outcome, we generated a patient mutation knock‐in mouse model utilizing a CRISPR/Cas strategy. For this, we introduced a missense point mutation at DNA position 590 G > T of the *Taz* gene, which causes a glycine to valine transition at position 197 and corresponds to a known patient mutation (Dudek *et al*, 2013; Fig EV1A). The mutant male mice were sterile with significantly smaller testis size (Fig 1A) and could not be utilized for generating homozygous litters. Thus, heterozygous females were paired with WT male mice to generate hemizygous male mice. Hence, for this study, we utilized exclusively hemizygous males (TAZ^G197V^) mice. Heterozygous females did not display reduced amounts of TAZ protein levels compared to the WT control (Fig EV1B). Body weight seems to be stabilized over time, opposite to male mice (Fig EV1C). Yet, the hemizygous male littermates were consistently born with smaller body size and lower body weight compared to their WT counterparts (Fig 1A), which persisted with age (Fig 1B). The mutant TAZ^G197V^ protein could not be detected in heart samples (Fig 1C) or other organs of the mutant male mice (Fig EV1D). About 40% of the liveborn TAZ^G197V^ male mice died within the first 20 days (Fig 1D).

BTHS patients display neutropenia as a clinical feature (Barth *et al*, 1983). Therefore, we analyzed blood cells in TAZ^G197V^ mice. As expected, the TAZ^G197V^ mice displayed a significantly lower number of circulating neutrophils and white blood cells (WBC; Fig 1E), while no changes were observed for total red blood cell (RBC) compared to WT mice. To address cardiac function, we performed echocardiographic analysis at 8‐, 14‐, and 20‐weeks of age. Until age of 14 weeks, no significant phenotypes were apparent for various parameters. Yet, at 20 weeks a notable decrease was detected for stroke volume, cardiac output (Fig EV1E and F), ejection fraction, and fractional shortening (Fig 1F and G) in the TAZ^G197V^ mice compared to the WT controls. Accordingly, TAZ^G197V^ mutant mice developed a progressive heart failure as they age. Echocardiographic evaluation of the deformation revealed heart failure due to systolic impairment. At 20 weeks of age, a significant impairment in longitudinal systolic strain was observed in the TAZ^G197V^ mice (Fig 1H). When the other two axes were evaluated, radial and circumferential systolic strain also showed impairment of the inotropic capacity of the heart. In contrast, the diastolic strain remained unchanged throughout the monitoring (Fig EV1H–J). A significant decrease was also measured in the left ventricle posterior wall (LVPW) thickness during systole, suggesting that the heart progresses toward myocardial remodeling (Fig EV1G). Accordingly, histochemical analysis revealed intercellular, interstitial, and endocardial fibrosis in TAZ^G197V^ heart samples in 20‐week‐old mice (Fig 1I). Consistent with myocardial dysfunction, expression of genes linked to cardiac failure such as *Nppa* (natriuretic peptide A) and *Nppb* (natriuretic peptide B) were markedly upregulated in the TAZ^G197V^ mice heart samples (Fig 1J).

### Defective mitochondrial functionality in TAZ^G197V^ mice

**Figure 2:**
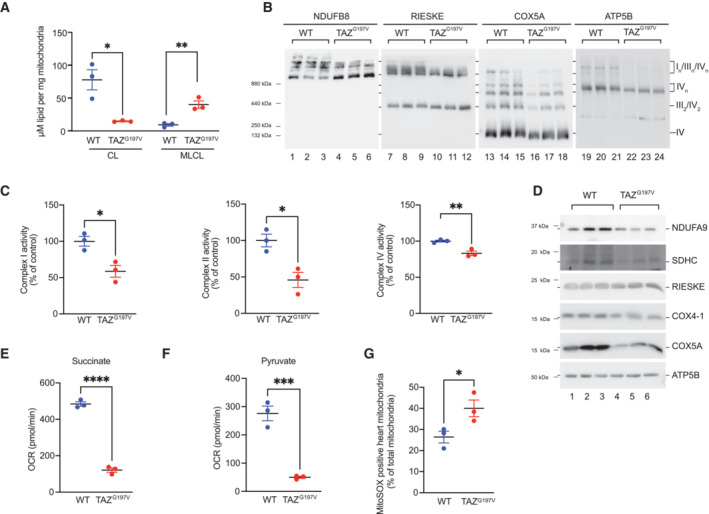
Molecular analysis of the TAZ ^G197V^ mouse AMass spectrometric analysis of Cardiolipin and Monolysocardiolipin in WT and TAZ^G197V^ mouse heart. Mean ± SEM, *n* = 3, unpaired *t*‐test: *P < 0.05, **P < 0.01.BWestern blot analysis of BN‐PAGE separated protein complexes from digitonin solubilized heart mitochondria (12‐week‐old mice).CQuantification of the activity of mitochondrial complexes, I, II, and IV in isolated mitochondria from 12‐week‐old mouse heart. Mean ± SEM, *n* = 3, unpaired *t*‐test: *P < 0.05, **P < 0.01.DSteady state protein levels of isolated mitochondria from 12‐week‐old mouse hearts.E, FOxygen consumption rate of isolated heart mitochondria from 12‐week‐old mice using succinate (E) and pyruvate (F) as substrates. Mean ± SEM, *n* = 3, unpaired *t*‐test: ***P < 0.001, ****P < 0.0001.GFlow cytometric analysis of isolated mitochondria using MitoSOX Red staining. Mean ± SEM, *n* = 3, unpaired *t*‐test: *P < 0.05. Source data are available online for this figure.

**Figure EV2:**
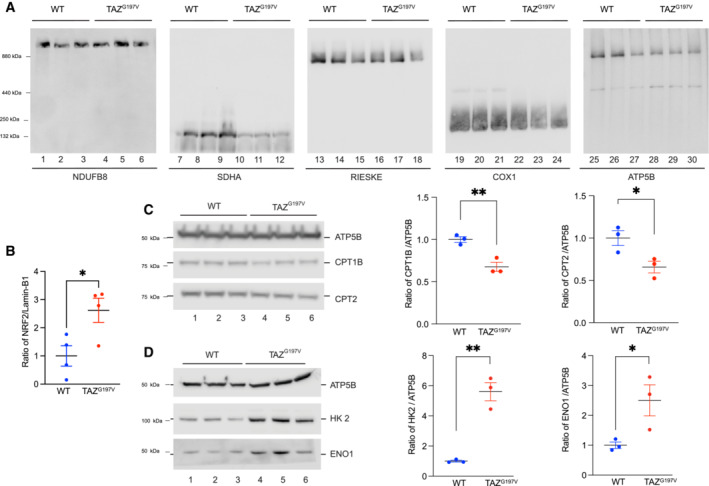
Molecular analysis of the TAZ^G197V^ mouse BN‐PAGE analysis of heart mitochondria solubilized in 1% DDM (12‐week‐old mice).Quantification of NRF2 levels on Western blot normalized to Lamin‐B1. Mean ± SEM, *n* = 4, unpaired *t*‐test: *P < 0.05.Western blot analysis of isolated mouse heart (20‐week‐old mice) mitochondria (left) and quantification (right) of CPT1 and CPT2 protein levels normalized to ATP5B. Mean ± SEM, *n* = 3, unpaired *t*‐test: *P < 0.05, **P < 0.01.Western blot analysis as in a (left) and quantification of HK2 and ENO1 normalized to ATP5B. Mean ± SEM, *n* = 3, unpaired *t*‐test: *P < 0.05, **P < 0.01.

Defects in the synthesis of CL due to loss of TAZ function result in formation of monolysocardiolipin (MLCL) and reduced mature CL levels. To confirm this phenotype in the TAZ^G197V^ BTHS mouse model, we analyzed CL and MLCL by mass spectrometry. In comparison to the WT mouse hearts, TAZ^G197V^ heart samples displayed the typical defects in CL remodeling with lower levels of CL and accumulation of MLCL and a shift of CL and MLCL toward more saturated species (Fig 2A, Dataset EV1). Next, we addressed the structural organization of the oxidative phosphorylation system in mitochondria isolated from TAZ^G197V^ mice heart. Membranes from purified mitochondria of the respective heart tissues were solubilized in the mild detergent digitonin and protein complexes analyzed by Blue‐Native gel electrophoresis (BN‐PAGE). While supercomplexes of complexes I, III, and IV were detected in the high molecular weight range in WT samples, these supercomplexes were reduced in the TAZ^G197V^ samples (Fig 2B). Complex II levels were significantly reduced in TAZ^G197V^ samples analyzed by BN‐PAGE and solubilized in n‐Dodecyl‐β‐D‐maltoside (DDM; Fig EV2A). The enzymatic activities of complexes I, II, and IV were also significantly reduced in TAZ^G197V^ mice heart mitochondria (Fig 2C). This could be attributed to a marked decrease of subunits of complex I, II, and IV as assessed by steady state protein analysis of NDUAFB9, SDHC, COX4‐1, and COX5A in the mutant mice mitochondria compared to the WT control. Conversely, no changes were observed for the protein level of Rieske and ATP5B (Fig 2D). These apparent reductions in respiratory chain abundance and activity were also reflected upon analysis of the respiration capacity using real‐time respirometry. A significant decrease in oxygen consumption was observed in the substrate‐dependent basal respiration with succinate (Fig 2E) and pyruvate (Fig 2F) in isolated mitochondria from TAZ^G197V^ heart samples compared to WT samples. As previous studies showed that structural reorganization of the respiratory chain correlated with less efficient energy coupling and generation of reactive oxygen species (ROS; Le *et al*, 2020; Liu *et al*, 2021), we measured ROS production in isolated mitochondria from WT and TAZ^G197V^ mouse heart using mitoSOX fluorogenic dye. Mitochondria from TAZ^G197V^ mouse heart displayed a significant increase in ROS generation compared to the WT control (Fig 2G). Moreover, we found increased NRF2 protein levels in TAZ^G197V^ mouse heart samples and elevated expression of NRF2‐regulated genes (e.g. NQO1, HMOX1, SRXN1, TXNRD1) further supporting an increase in ROS levels (Fig EV2B, Dataset EV2). In summary, we concluded that due to the lack of cardiolipin in the TAZ^G197V^ BTHS patient mutation mouse model, respirasome formation and abundance were significantly affected. This defect leads to reduced respiration capacity and elevated ROS levels.

### Reduced expression of fatty acid oxidation related genes in TAZ^G197V^ heart

The heart is metabolically flexible, switching between metabolic pathways based on available energy substrates to constantly maintain cardiac contractility (Karwi *et al*, 2018). Approx. 95% of cardiac ATP is derived by mitochondrial oxidative phosphorylation, while glycolysis provides the remaining 5% of the energy demand (Barth *et al*, 1983; Lopaschuk *et al*, 2021). The majority of the mitochondrial ATP production (≈40 to 60%) is fueled by fatty acid oxidation (FAO), while the remainder is driven by oxidation of pyruvate (from glucose and lactate), ketone bodies, and amino acids (Lopaschuk *et al*, 2021). Heart failure is associated with an uncoupling of cytosolic metabolic pathways from mitochondrial oxidative metabolism. Therefore, metabolic rewiring due to mitochondrial dysfunction is considered as key driver in heart failure (Karwi *et al*, 2018; Lopaschuk *et al*, 2021). Compromised mitochondrial oxidative phosphorylation was shown to increase reliance on glycolysis (Zhang *et al*, 2013) with FAO decreasing in failing heart (Bedi *et al*, 2016; Ho *et al*, 2017; Wende *et al*, 2017).

**Figure 3:**
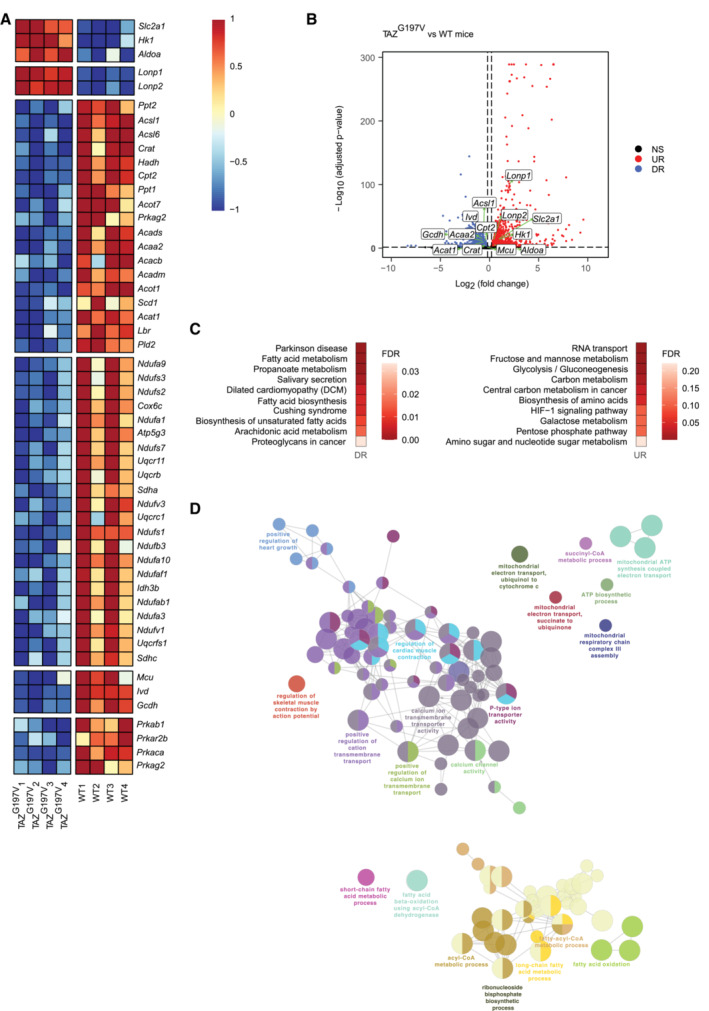
Gene expression profiling in TAZ^G197V^ mice heart Heatmap representation of normalized expression (centered and scaled) of selected genes involved in biological pathways relevant for the study, in 14‐week‐old WT and TAZ^G197V^ mice.Volcano plot of differentially expressed genes between 14‐week‐old WT and TAZ^G197V^ mice. X‐axis denotes fold change in expression (log_2_ scale), y‐axis denotes adjusted *P*‐value (negative log_10_ scale) for the analyzed genes in the data (each dot represents one single gene). Blue and red dots represent genes significantly downregulated (DR) and upregulated (UR), respectively, in the TAZ^G197V^ mice compared to WT mice. The cut‐offs used were: adjusted *P*‐value < 0.05 and fold change > 1.15 or < −1.15. Non‐significant (NS) genes are depicted in black. Selected genes involved in pathways relevant for the study are labeled in the plot.(Left) Over‐representation analysis of selected significantly downregulated genes in 14‐week‐old TAZ^G197V^ mice and (right) over‐representation analysis of selected significantly upregulated genes (left) in TAZ^G197V^ mice. Functional pathways derived from KEGG database, top 10 over‐represented (enriched) pathways were selected for visualization. Color indicates the enrichment FDR value (Benjamini–Hochberg procedure).ClueGO network visualization of groups of enriched functional pathways (biological processes) for the selected significantly downregulated genes in TAZ^G197V^ mice. The most significant pathways/terms among a group of pathways are highlighted in the network. The size of the nodes indicates the number of genes from the given list involved in that pathway.

We performed RNA sequencing (RNA‐seq) analysis from WT and TAZ^G197V^ mouse heart samples. In TAZ^G197V^ hearts, the expression profiles of genes related to FAO and glycolytic pathways were significantly altered. Differential gene expression analysis indicated a decrease in several FAO genes including the carnitine palmitoyl transferase 2 gene (Cpt2). There was also a decrease in expression of genes involved in mitochondrial electron transport chain, TCA cycle, and mitochondrial calcium transporter proteins. Moreover, several important glycolytic pathway genes including *Glut1*(*Slc2a1*), *Hk1*, and *Aldoa* were significantly upregulated (Fig 3A and B).

An over‐representation (pathway enrichment) analysis using terms from the KEGG pathway database revealed that pathways related to fatty acid metabolism, fatty acid biosynthesis, and biosynthesis of unsaturated fatty acids were enriched among genes with decreased expression in the mutant samples compared to the wild type (Fig 3C (left)). These observations were supported by a network level grouping and visualization of Gene Ontology biological process terms enriched among these downregulated genes, which indicated similar pathways as above and also provided an insight into how these pathways can be linked functionally, based on shared genes (Fig 3D). On the other hand, pathways such as glycolysis, gluconeogenesis, carbon metabolism, galactose metabolism, biosynthesis of amino acids and pentose phosphate pathways showed enrichment among genes with higher expression in the mutant samples (Fig 3C (right)).

### Altered fatty acid regulation and glycolysis in cardiac tissue of TAZ^G197V^ mice

**Figure 4:**
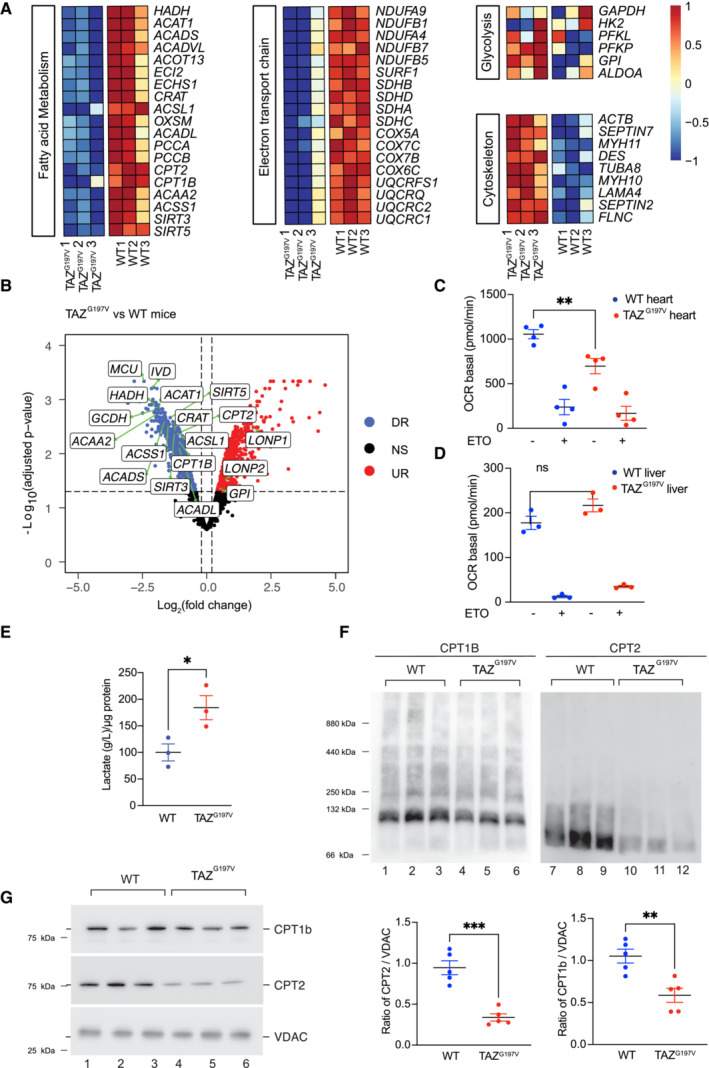
Reduced fatty acid oxidation in cardiac tissue of TAZG^197V^ mice AHeatmap showing normalized expression (centered and scaled) for selected proteins involved in biological pathways relevant for the study, in 14‐week‐old WT and TAZ^G197V^ mice.BVolcano plot of differentially expressed proteins between 14‐week‐old WT and TAZ^G197V^ mice. X‐axis denotes fold change in expression (log_2_ scale), y‐axis denotes adjusted *P*‐value (negative log_10_ scale) for analyzed proteins in the data (each dot represents one single protein). Blue and red dots represent proteins significantly downregulated (DR) and upregulated (UR), respectively, in the TAZ^G197V^ mice compared to WT mice. The cut‐offs used were: adjusted *P*‐value < 0.05 and fold change > 1.15 or < −1.15. Non‐significant (NS) proteins are depicted in black. Selected proteins involved in pathways relevant for the study are labeled in the plot.C, DReal‐time respirometry, oxygen consumption rate of isolated heart (C) and liver (D) mitochondria from (20‐week‐old mice) driven by palmitoyl/carnitine/malate/ADP. ETO, etomoxir. Mean ± SEM, *n* = 3, unpaired *t*‐test: **P < 0.01, ns (non‐significant).ELactate levels in heart tissue (20 weeks old mice). Mean ± SEM, *n* = 3, unpaired *t*‐test: *P < 0.05.FWestern Blot analysis of BN‐PAGE separated complexes from digitonin solubilized heart mitochondria (12‐week‐old mice).GSteady state protein levels of isolated heart mitochondria (12‐week‐old mice). CPT1B and CPT2 protein levels were normalized to VDAC. Mean ± SEM, *n* = 5, unpaired *t*‐test: **P < 0.01, ***P < 0.001. Source data are available online for this figure.

To define changes in fatty acid oxidation and glycolysis at the protein level, we performed a proteomic analysis of TAZ^G197V^ and WT mice heart mitochondria enriched fractions. Mitochondrial proteins were subjected to TMT6‐labelling followed by fractionation using high pH reversed‐phase chromatography (bRP) and further SPS‐MS3‐mediated fragment ion detection in an Orbitrap analyzer. The proteomic data were analyzed using limma software package to identify differences between TAZ^G197V^ and WT mitochondria. A heatmap representation of protein expression profiles from this proteomic analysis revealed reduced abundance of fatty acid oxidation‐related proteins such as CPT1B, CPT2, CRAT, ACADVL, HADH along with mitochondrial electron transport chain proteins, cytoskeletal proteins, and mitochondrial calcium transporters (Fig 4A and B). Conversely, glycolytic proteins, which were still identified in the mitochondria‐enriched preparation, such as HK2, ALDOA, GAPDH etc. were increased in the mutant samples (Fig 4A).

Accordingly, we measured fatty acid‐driven mitochondrial respiration by real‐time respirometry using isolated heart mitochondria. The basal respiration using palmitatoyl/carnitine/malate/ADP as a substrate was compromised in isolated mitochondria from TAZ^G197V^ heart samples (Fig 4C). Addition of etomoxir (ETO), an inhibitor of CPT1, served as a negative control, indicating that the measured respiration was specific to fatty acid oxidation. Yet, no significant difference in the fatty acid oxidation driven respiration was apparent in isolated mitochondria from TAZ^G197V^ liver (Fig 4D). Since we observed increased expression of glycolytic genes in the RNAseq analysis, we determined the lactate levels in heart samples from TAZ^G197V^ and WT mice. Indeed, we observed a significant increase in the amount of lactate production in the TAZ^G197V^ mouse hearts compared to control (Fig 4E) indicating that the mutant hearts display an increased glycolytic metabolism.

To assess the levels of CPT1B and CPT2 in their native complex in mitochondria, we solubilized mitochondria and monitored protein complexes by BN‐PAGE. The amount of CPT1B‐ and CPT2‐containing complexes were clearly reduced in TAZ^G197V^ heart mitochondria compared to WT mitochondria (Fig 4F). Moreover, steady state protein analysis presented significantly diminished protein levels of CPT1B and CPT2 in TAZ^G197V^ heart mitochondria (Fig 4G). Since we observed a decline in cardiac function especially in 20‐week‐old mice, we addressed whether the decrease of FAO proteins was retained. Indeed, a significant decrease in steady state levels of CPT1B and CPT2 was apparent in 20‐week‐old TAZ^G197V^ heart mitochondria (Fig EV2C). Moreover, a notable increase in glycolytic protein levels such as HK2 and ENO1 was evident in these samples (Fig EV2D) indicating increased glycolytic function.

### iPSC‐cardiomyocytes recapitulate loss of fatty acid oxidation

**Figure 5:**
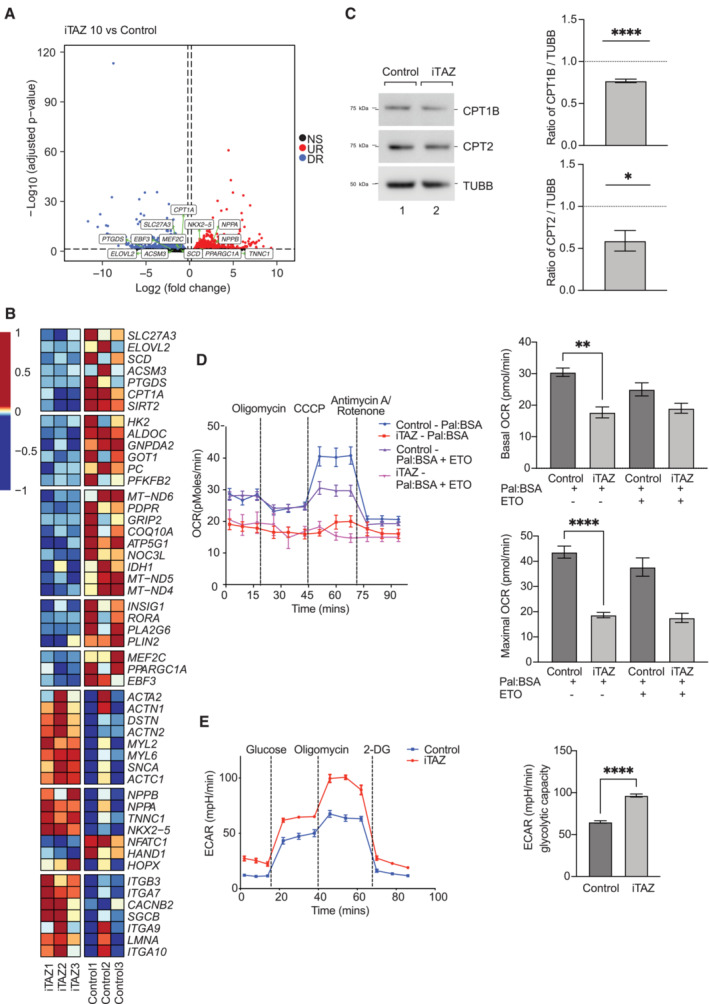
iPSC‐cardiomyocytes display reduced fatty acid oxidation Volcano plot presentations of differentially expressed genes, control versus iTAZ iPSC samples. X‐axis, fold change in expression (log_2_ scale); y‐axis, adjusted *P*‐value (negative log_10_ scale) for analyzed genes (each dot represents one single gene). Blue and red dots represent genes significantly downregulated (DR) and upregulated (UR), respectively, in the iTAZ samples compared to control. The cut‐offs used were: adjusted *P*‐value < 0.05 and fold change > 1.15 or < −1.15. Non‐significant (NS) genes are depicted in black. Selected genes involved in pathways relevant for the study are indicated in the plot.Heatmap showing normalized expression (centered and scaled) of selected genes involved in biological pathways relevant for the study, in control and iTAZ samples.Western blot steady state protein levels of CPT1B and CPT2. Values were normalized to β‐tubulin in control and iTAZ iPSC‐cardiomyocyte. Mean of WT values was set to 1. Mean ± SEM, *n* = 5, unpaired *t*‐test: *P < 0.05, ****P < 0.0001.Real‐time respirometry. Left, Oxygen consumption measured as basal, and upon addition of oligomycin, CCCP, and antimycin/rotenone using palmitate‐BSA as substrate, ETO, etomoxir. Right, quantification of basal and maximal respiration. Mean ± SEM, *n* = 4, unpaired *t*‐test: **P < 0.01, ****P < 0.0001.Extracellular acidification rate of control and iTAZ iPSC‐cardiomyocytes. Basal state and upon sequential addition of glucose, oligomycin and 2 deoxyglucose (2‐DG). Right, quantification of glycolytic capacity. Mean ± SEM, *n* = 6, unpaired *t*‐test: ****P < 0.001. Source data are available online for this figure.

BTHS patient‐induced pluripotent stem cell (iPSC)‐derived cardiomyocytes, harboring the corresponding amino acid exchange G197V as the described mouse, display alterations in morphology and sarcomeric structure, impaired respiration, and inability to adapt to hypoxic conditions (Dudek *et al*, 2016; Chowdhury *et al*, 2018). To assess metabolic reorganization in the context of a relevant human cardiac cell system, we used these BTHS patient iPSC‐derived cardiomyocytes. RNA‐seq analyses in healthy control versus patient iPSC‐derived cardiomyocytes (iTAZ cardiomyocyte) samples revealed similar alterations in the gene expression profile as observed in mice hearts. Genes related to FAO pathways, including *CPT1A*, were significantly low in expression in the patient (iTAZ) cardiomyocytes, while cardiac failure markers such as *NPPA*, *NPPB*, *NKX2‐5* were upregulated in the same (Fig 5A). Additionally, genes related to the TCA cycle, electron transport chain, lipid metabolism, mitochondrial biogenesis, and actin cytoskeleton regulation were downregulated as shown in a heat map presentation (Fig 5B). In contrast, we found that genes involved in dilated cardiomyopathy and hypertrophy displayed an upregulation in iTAZ cardiomyocytes compared to the control. To confirm the relevance of these observations at the protein level, we performed Western blot analysis. Indeed, we found decreased levels of CPT1B and CPT2 in the patient derived iTAZ cardiomyocytes in agreement with the observations in the mutant mouse heart (Fig 5C). To further address whether the observed decrease of CPTs in the iTAZ cardiomyocytes was reflected in the fatty acid‐driven oxygen consumption rate, we performed real‐time respirometry in live cardiomyocytes using palmitate‐BSA as a substrate. Etomoxir (ETO), which inhibits CPT function, was used as a control. These analyses revealed diminished oxygen consumption rate in iTAZ cardiomyocytes. In the absence of ETO, iTAZ cardiomyocytes presented a significantly reduced basal and maximal respiration rate compared to the healthy‐control (isWT1.14) cardiomyocytes (Fig 5D). Next, we assessed the glycolysis rate in the cardiomyocytes. Although no significant changes in expression of glycolytic genes were apparent in RNAseq analysis, the iTAZ cardiomyocytes displayed a marked increase in the maximal glycolytic capacity upon stimulation in a glycolytic stress test (Fig 5E).

**Figure EV3:**
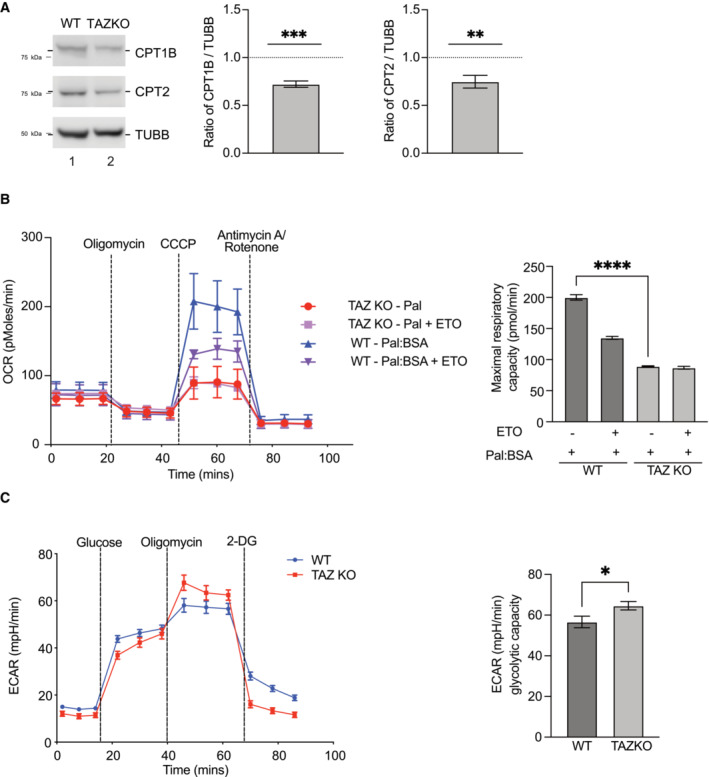
TAZ‐deficient MEF cells display reduced fatty acid oxidation Western blot analysis (left) and quantification (right) of CPT1 and CPT2 protein levels in WT and TAZKO MEF cell lysate normalized to β‐tubulin. Mean ± SEM, *n* = 4, unpaired *t*‐test: **P < 0.01, ***P < 0.001.Oxygen consumption rate of WT and TAZ MEF cells in media supplemented with palmitate‐BSA, basal, oligomycin, CCCP, and antimycin/rotenone treated (left). ETO, etomoxir. Quantification of maximal respiratory capacity (right). Mean ± SEM, *n* = 3, unpaired *t*‐test: ****P < 0.0001.Extracellular acidification rate, presentation of basal rate, addition of glucose, oligomycin, and 2 deoxyglucose (2‐DG). Quantification of Glycolysis and Glycolytic capacity of the cells is presented on the right. Mean ± SEM, *n* = 6, unpaired *t*‐test: *P < 0.05.

To further support these observations, we analyzed knockout (KO)‐TAZ mouse embryonic fibroblasts (MEFs) in which we previously observed metabolic similarities to patient‐derived cardiomyocytes (Chowdhury *et al*, 2018). Similar to the cardiomyocytes, TAZ KO‐MEFs displayed decreased protein levels of CTP1B and CPT2 (Fig EV3A). Real‐time respirometry under conditions of FAO revealed decreased OCR (Fig EV3B) and a glycolytic stress test also showed increased glycolysis (Fig EV3C) in TAZ KO‐MEFs compared to the control. Accordingly, knock out MEF displayed similar metabolic alterations as observed in cardiomyocytes and mice heart. Hence, we conclude that patient derived iPSC‐cardiomyocytes, mutant mice, and knock out MEF cells display reduced mitochondrial fatty acid oxidation accompanied with increased glycolysis.

### Elevated glycolytic ATP levels attenuate AMPK signaling

**Figure 6:**
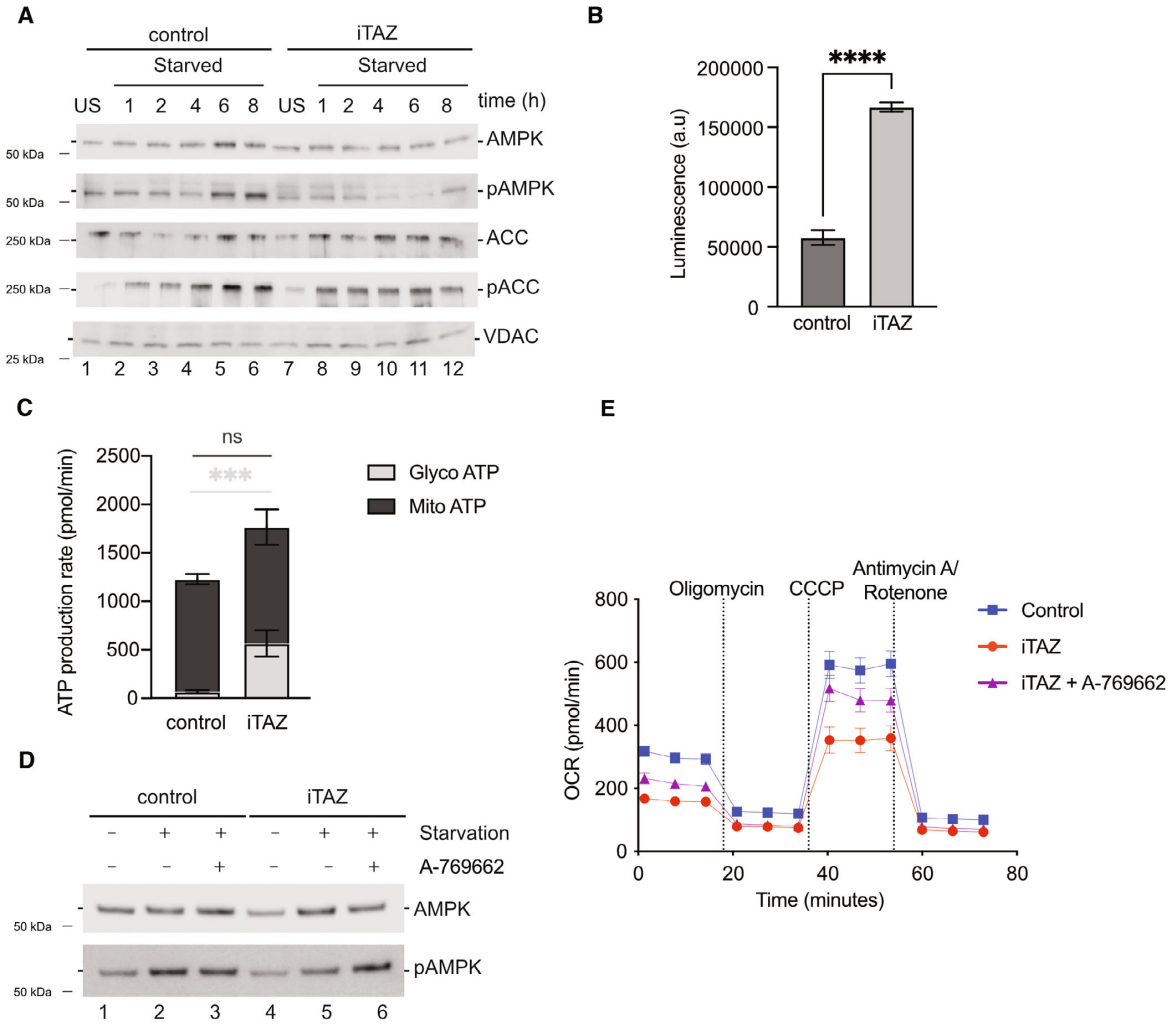
Altered AMPK signaling Western blot analysis of steady state protein levels of untreated (US) and cells starved (S) for indicated times.Total ATP amounts displayed as arbitrary luminescence units. Mean ± SEM, *n* = 3, unpaired *t*‐test: ****P < 0.0001.Glycolytic and mitochondrial ATP production measured using a real‐time ATP rate assay. Mean ± SEM, *n* = 3, unpaired *t*‐test: ***P < 0.001.Western blot analysis of steady state AMPK and pAMPK protein levels of starved and/or A769662 (AMPK activator) treated cells as indicated.Real‐time respirometry. Oxygen consumption rate of indicated iPSC‐cardiomyocytes A769662 treated or not Mean ± SEM, *n* = 3. Source data are available online for this figure.

**Figure EV4:**
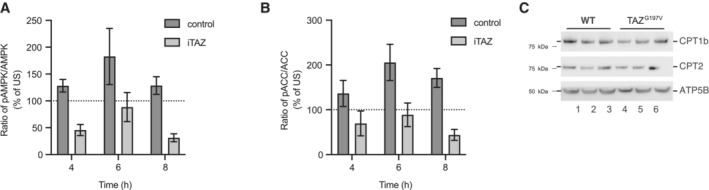
Altered AMPK signaling in iPSC‐cardiomyocytes and mice Quantification of pAMPK/AMPK and.pACC/ACC ratio, under starvation conditions at indicated hours for control and iTAZ iPSC‐cardiomyocytes. Unstarved ratio set as 100%. Mean ± SEM, *n* = 3.CPT1 and CPT2 protein levels from isolated heart mitochondria of 24‐week‐old mice. Mice were treated with A‐769662 (AMPK activator) for 6 weeks prior to analysis.

**Figure EV5:**
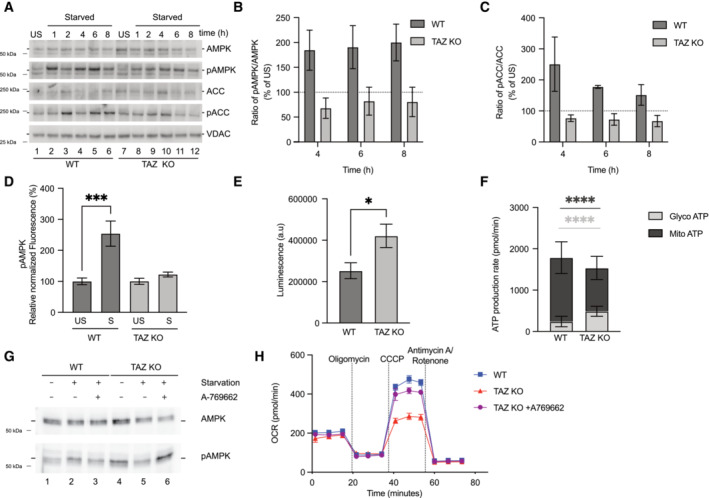
Altered AMPK signaling in MEF cells AWestern blot analysis of steady state protein levels of WT and TAZ KO MEF cell lysates. Cells were subjected to starvation for indicated times or left unstarved.B, CQuantification of pAMPK/AMPK and (C) pACC/ACC ratio, under starvation conditions at indicated hours for WT and TAZ KO MEF cells. Unstarved ratio set as 100%. Mean ± SEM, *n* = 3.DAmount of pAMPK in WT and TAZ KO MEF cell lysate. Cells were subjected to 8 h of starvation. Fluorescence signal was normalized to total protein amount. Mean ± SEM, *n* = 3, unpaired *t*‐test: ***P < 0.001.EQuantification of total ATP amounts (arbitrary luminescence units) in WT and TAZ KO MEF cell lysate. Mean ± SEM, *n* = 3, unpaired *t*‐test: *P < 0.05.FReal‐time ATP rate assay to measure ATP production rate (glycolytic and mitochondrial). Mean ± SEM, *n* = 3, unpaired *t*‐test: ****P < 0.0001.GWestern blot analysis of steady state protein levels of cell lysates from cells treated as indicated. A‐769662 (AMPK activator).HReal‐time respirometry, basal oxygen consumption rate (OCR), oligomycin, CCCP and antimycin/rotenone treated for WT and TAZ KO (non‐treated and A‐769662 treated). Mean ± SEM, *n* = 3.

To define the mechanism underlying downregulation of the fatty acid oxidation pathway and decline of cardiac function, we investigated pathways which were directly involved in transcriptional regulation of CPTs. Transcriptomic analysis of mice hearts showed a significant reduction of AMPK subunits such as Prkab1, Prkag2 (Fig 3A). Similarly, proteomic analysis of mice hearts displayed diminished abundance of AMPK subunits PRKAG1, PRKACA etc. Additionally, transcriptome analysis of the iTAZ cardiomyocytes revealed a downregulation of PGC1α (*PPARGC1A*) compared to the control (Fig 5B). PGC1α is under the control of AMPK activation. Activation of AMPK leads to phosphorylation of PGC1α, which is a direct transcriptional coactivator for a variety of transcription factors involved in biological responses such mitochondrial biogenesis, glucose/fatty acid metabolism, heart development etc. PGC1α directly regulates the expression of CPT (Liang & Ward, 2006). Thus, we hypothesized that the ‘AMPK‐PGC1α’‐mediated regulation of CPT‐1 was responsible for the phenotypes observed in TAZ‐deficient cells. To this end, we subjected the iPSC‐derived cardiomyocytes (control and iTAZ) to glucose starvation to activate the AMPK pathway that is known to trigger fatty acid oxidation in cells (Ren & Shen, 2019). Upon starvation, we observed an increase in the phosphorylated AMPK (pAMPK) form over time, in the healthy control cardiomyocytes. However, in iTAZ cardiomyocytes the phosphorylated form of AMPK (pAMPK) did not increase (Figs 6A and EV4A). The activated AMPK phosphorylates and thereby inhibits the acetyl‐CoA carboxylase (ACC) to increase rates of myocardial fatty acid oxidation (Mihaylova & Shaw, 2011). In agreement, increased phosphorylation of ACC (pACC) was apparent in control cardiomyocytes but a substantially reduced response was observed in iTAZ cardiomyocytes (Figs 6A and EV4B). Similar results were obtained for TAZ‐KO MEF. A gradual increase of pAMPK and pACC over time was found in wild type MEF upon starvation that were not apparent in the TAZ‐KO MEF (Figs EV5A and 4B and C). This observation was confirmed with an ELISA based AMPK assay (Fig EV5D).

The observed lack of AMPK activation in TAZ mutant cells led us to investigate the reason for this defect. Under physiological conditions, an increase in the AMP:ATP ratio triggers AMPK activation (Lin & Hardie, 2018). Therefore, we assessed total ATP levels in mutant cells. To our surprise, we noticed a significant increase in the ATP levels in iTAZ cardiomyocytes compared to the control (Fig 6B). A similar observation was made in TAZ‐KO MEF (EV5E). Since oxidative phosphorylation appeared to be reduced in the mutant (Fig 4D), we investigated the source of ATP in the cells. To this end, we performed real‐time ATP rate assays to measure the rate of ATP production from the two key energetic pathways i.e glycolysis and mitochondrial oxidative phosphorylation. Interestingly, while we found no difference between control and iTAZ cardiomyocytes regarding their mitochondrial ATP production rates, a significant increase was apparent for the cytoplasmic, glycolytic ATP production in the iTAZ cardiomyocytes (Fig 6C). A corresponding experiment in the MEF model revealed an increased amount of glycolytic ATP accompanied with decreased levels of mitochondrial ATP production in the TAZ‐KO (Fig EV5F). We concluded that the greater levels of ATP observed in the TAZ‐deficient models originated from increased glycolysis and that this rendered AMPK inactive to induce fatty acid oxidation driven respiration. To support this hypothesis, we subjected the cardiomyocytes (WT and iTAZ) to a treatment with A769662, an allosteric activator for the AMPK pathway. A769662 activates AMPK by mimicking the effects of AMP both allosterically and by inhibiting dephosphorylation of AMPK. Accordingly, A769662 treatment has been shown to inhibit fatty acid synthesis and increase FAO in primary rat hepatocytes as well as lowers blood glucose in rats (Cool *et al*, 2006). Upon A769662 treatment of iTAZ and control cardiomyocytes, we observed an increase in AMPK phosphorylation in iTAZ cardiomyocytes (Fig 6D) In a similar experiment on MEF, an increase of pAMPK was observed in TAZ KO MEF upon A769662 treatment, which was comparable to WT (Fig EV5G). The activation of AMPK pathway was consequently reflected when we measured fatty acid‐driven respiration by real‐time respirometry, where addition of A769662 increased oxygen consumption of iTAZ mitochondria compared to the untreated control (Fig 6E) which was similarly observed in the MEFs (Fig EV5H).

**Figure 7:**
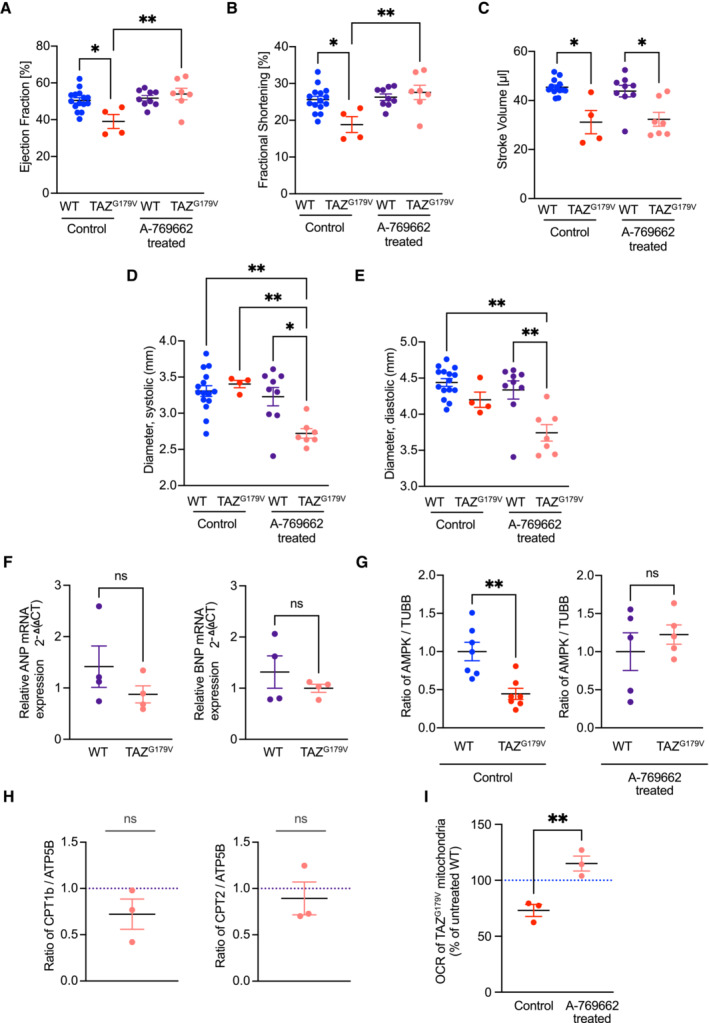
Effect of AMPK activator on TAZ^G197V^ mice A–EEjection fraction from short axis expressed in %, (B) Fractional shortening in %, (C) Stroke volume in μl (D) Systolic diameter in mm and (E) Diastolic diameter in mm. Mean ± SEM, *n* = 15 (WT control); 4 (TAZ^G197V^ control); 9 (WT treated); 7 (TAZ^G197V^ treated), 2Way ANOVA: *P < 0.05, **P < 0.01.FGene expression addressed by qPCR of ANP (left) and BNP (right) in 24‐week‐old WT and TAZ^G197V^ mice heart upon 6‐week treatment with A769662. Mean ± SEM, *n* = 4, unpaired *t*‐test: ns (non‐significant).GQuantification of steady state levels of AMPK normalized to beta tubulin in tissue lysates from 24‐week‐old WT and TAZ^G197V^ mice heart from untreated control or following a 6‐week treatment with A769662. The mean of the WT values was set to 1. Mean ± SEM, *n* = 7 (control); 5 (treated), unpaired *t*‐test: **P < 0.01.HQuantification of steady state levels of CPT1B and CPT2 normalized to ATP5B in tissue lysate of 24‐week‐old WT and TAZ^G197V^ mice heart following 6‐week treatment with A769662. The mean of the WT values was set to 1. Mean ± SEM, *n* = 3, unpaired *t*‐test: ns (non‐significant).IReal‐time respirometry, oxygen consumption rate, of isolated heart mitochondria from 24‐week‐old TAZ^G197V^ mice, from untreated control or following a 6‐week treatment with A769662, driven by palmitoyl/carnitine/malate/ADP. The OCR of the untreated WT samples was taken as 100%. Mean ± SEM, *n* = 3, unpaired *t*‐test: **P < 0.01. Source data are available online for this figure.

Based on the finding in cellular systems, where we observed that pharmacologic activation of AMPK reversed the switch from fatty acid‐driven respiration to glycolysis in TAZ‐deficient cells, we investigated the effect of A769662 on the patient mutation mice and whether it affected cardiac function. For this, 18 weeks old WT and TAZ^G197V^ mice were injected intraperitonially with A769662 at a dose of 30 mg/kg daily for 6 weeks. Vehicle treated mice served as a control group. As mentioned before, the mutant mice did not manifest any pathological cardiac outcomes until the age of 20 weeks (Fig 1F). Therefore, we started the administration of the compound at an age with no significant difference in the cardiac function of mutant mice, i.e. at the age of 18 weeks. While TAZ^G197V^ mice displayed reduced ejection fraction and fractional shortening compared to the wild type mice at the age of 24 weeks, mutant mice treated with A769662 showed preserved ejection fraction (Fig 7A) and fractional shortening (Fig 7B). Still, the stroke volume (Fig 7C) appeared to be decreased to similar levels in the A769662 treated and vehicle control group. Additionally, significant decrease in both the systolic (Fig 7D) and diastolic (Fig 7E) diameter were observed in the treated group compared to the vehicle control group, hinting that the improvement of the ejection fraction and fractional shortening was mostly due to a geometric change of the heart in these animals.

Unlike untreated TAZ^G197V^ mice (Fig 1J), mRNA expression of *Nppa* and *Nppb* in the A769662 administered mice remained at the physiological range observed in wild type controls (Fig 7F). Analysis of AMPK protein levels revealed a significant decrease in untreated TAZ^G197V^ mutant mice which was increased to wild type level upon A769662 treatment (Fig 7G). At the protein level, CPT1B and CPT2 remained similar to wild type when A769662 was administered to the mutant mice (Figs 7H and EV4C). In agreement, FAO‐driven respiration measured in mouse heart mitochondria was fully recovered in the treated TAZ^G197V^ mutant animals (Fig 7I). Accordingly, upon treatment with A769662, fatty acid metabolism can be triggered in an AMPK pathway dependent manner in the TAZ‐deficient models, which in turn can rescue the cardiac dysfunction of the patient mutation mice. Thus, emphasizing the potential therapeutic implications for AMPK activation in BTHS patients and associated cardiac debilitation.

## Discussion

BTHS is a multisystem disorder with cardiomyopathy having a major role in manifestation and progression of the disease. To study and to develop therapeutic strategies against BTHS, relevant animal model systems are a key requirement. Most BTHS mice models that have been reported previously do not closely match the human BTHS genetics (Acehan *et al*, 2011; Ren *et al*, 2019; Wang *et al*, 2020). A patient *knock‐in* mouse (Taz^D75H^) has been reported, yet without study of the cardiac outcome (Edwards *et al*, 2017). The most extensively studied BTHS mouse model is a doxycycline‐induced short hairpin RNA Taz KD mouse, in which administration of doxycycline is required to achieve 80–90% depletion of TAZ protein. With a preserved ejection fraction and an age dependent progression of diastolic dysfunction, this mouse model reflects more the HFpEF patient population. Major limitations of this model are, a high interanimal variability, and the continuous need of doxycycline treatment, which by itself impacts mitochondrial function (Moullan *et al*, 2015) and metalloprotease activity (Golub *et al*, 1998). Two studies reported on *Taz* floxed mouse models (Wang *et al*, 2020; Zhu *et al*, 2021). In both cases, exons 5–10 of the *Taz* gene were flanked by LoxP sites which were used to generate *Taz* gKO as well as cardiac specific cKO mice. Mouse strain dependent perinatal lethality of the *Taz* gKO mice and low body weight limits the mouse model for studying *Taz* function and CL remodeling to understand BTHS cardiac dysfunction (Wang *et al*, 2020, 2023; Liu *et al*, 2021) Nevertheless, the tissue specific deletion of Taz revealed important aspects of BTHS, including the development of dilated cardiomyopathy and substantially reduced fractional shortening (Wang *et al*, 2020). The cardiac specific mutation, however, does not reflect heart failure as a systemic disease. In this study, we have generated a CRISPR‐Cas mediated BTHS patient missense mutation (G197V) *knock‐in* mouse model, which shows progressive systolic dysfunction, reflecting patients within their first weeks of life. Moreover, the mice present additional pathological symptoms including increased cardiac fibrosis, male infertility, neutropenia, stunted growth and reduced survival. The missense mutation in this model results in a loss of TAZ protein, due to which, it recapitulates the established molecular, biochemical, and mitochondrial defects that have been shown previously in several studies (Dudek *et al*, 2013, 2016, 2019, 2020).

Interestingly, proteomic and transcriptomic analysis of the hearts of the mouse model revealed attenuation of FAO pathways and concomitantly increased glycolytic gene expression which direct toward cardiac dysfunction. It is a well‐studied and accepted phenomenon that cardiac FAO is decreased in heart failure (Huss & Kelly, 2005; Neubauer, 2007; Ingwall, 2009), while its energy metabolism shifts toward glycolysis as a source of energy (Zhabyeyev *et al*, 2013; Zhang *et al*, 2013). A study, examining a TFAM knockout mouse model, showed this switch not to be a compensatory mechanism, but rather aggravating the phenotype. It is worth mentioning however, that this study showed no increase in overall ATP levels (Hansson *et al*, 2004). BTHS patients have been found to display blunted FAO and a consequent cardiac dysfunction (Cade *et al*, 2019, 2021). Moreover, in isolated cardiac mitochondria from *TazKD* mice and from cardiac‐specific *Tafazzin* deficient mice, mitochondria showed a significant repression of state 3 (ADP‐stimulated) respiration rates when respiring through the substrates palmitoylcarnitine and malate (Le *et al*, 2020; Zhu *et al*, 2021). In addition, increased glycolysis and elevated lactate levels in individuals with BTHS have been found (Ferri *et al*, 2013). Another study in isolated neonatal cardiac myocytes from *TazKD* mice demonstrated increase in ECAR rates suggestive of elevated glycolysis and decreased glucose oxidation (Powers *et al*, 2013). Recently, the analysis of myocardial tissue from BTHS patients revealed a reduction of long‐chain fatty acid oxidation enzymes (Chatfield *et al*, 2022). Here, we report a dysregulation of CPT1B and CPT2 by the AMPK signaling pathway. Similar to the observation in BTHS patients, we found a significant reduction of fatty acid oxidation enzymes in mutant mice. In the BTHS mutant mouse model established here and in patient iPSC‐cardiomyocytes, AMPK signaling is insufficient to activate PGC1α‐mediated transcription of the CPTs and Sirt3, which are directly involved in beta oxidation. PGC1α, on the other hand is self‐regulatory (Rius‐Pérez *et al*, 2020). The inactivity of the AMPK pathway is based on elevated ATP levels in the mutant. However, an increase in cellular ATP, in spite of diminished OXPHOS in Taz mutants could be explained by dissecting the ATP source in the cellular system. The boost in total ATP could clearly be attributed to increased glycolysis. Yet, AMPK activation has also been shown to promote glycolysis and glucose uptake (Taylor *et al*, 2008; Wu *et al*, 2013).

We delineated whether the enhancement of cardiac FAO alleviated the observed cardiomyopathy. To this end, we used A769662, an allosteric AMPK activator which has previously been shown to restore FAO in an AMPK dependent manner (Ducommun *et al*, 2014; Foretz *et al*, 2018). A restoration of AMPK signaling and consequently respiration via FAO in patient derived cardiomyocytes upon A769662 treatment was observed. In addition, a treatment of the BTHS mouse model with A769662 stabilized cardiac function, which in the non‐treated mice declined gradually over time. While we found improved ejection fraction and fractional shortening upon treatment, the stroke volume remained reduced. This finding is in part explained by a decrease in systolic and diastolic diameter of the heart. In agreement with this, mRNA levels of ANP, BNP, protein levels of CPTs and FAO‐driven oxidative phosphorylation were brought back to control level. Interestingly, while under starvation conditions in MEF cells and iPSC‐derived cardiomyocytes we observed alterations in the pAMPK/AMPK ratios, in mouse heart (unstarved) the total AMPK levels were reduced. Treatment with the AMPK activator fully recovered AMPK amounts to the physiological levels in the mutant mice whereas in the cellular systems the pAMPK/AMPK ratio were normalized.

We concluded, that AMPK activation is a potential means for treating cardiac failure in BTHS context. Our findings provide a mechanistic explanation how metabolic reorganization leads to cardiac dysfunction in BTHS patients and a provide potential therapeutic approach to mitigate the development and progression of BTHS‐related cardiomyopathy.

## Materials and Methods

### Mice generation

Mice were maintained and the experiments on them were performed according to the guidelines from the German Animal Welfare Act and approved by the *Landesamt für Verbraucherschutz und Lebensmittelsicherheit*, Niedersachsen, Germany (AZ: 33.9‐42502‐04‐15/1991). TAZ^G197V^ mice were generated through CRISPR/Cas9 based genome engineering. C57BL/6N donor female mice were super ovulated to obtain zygotes for microinjection. Pronuclear injection with a mix containing the CAS9 enzyme, sgRNA targeting the TAZ locus and a single stranded oligonucleotide DNA repair template was carried out using micro manipulators. Embryos were then surgically transferred intoC57BL/6N pseudo pregnant female mice. Edited founders were identified by Sanger sequencing from ear biopsies. Mice carrying the desired modification events were crossed with C57BL6/J for two generations to ensure germline transmission and eliminate any possible mosaicism. Heterozygous animals with the same modification were then mated to generate homozygous offspring. Subsequent genotypings were carried out by PACE (PCR Allele Competitive Extension) fluorescent endpoint genotyping.

### Cell culture work

#### Mouse Embryonal Fibroblasts (MEF)

DMEM supplemented with 10% [v/v] FBS was used to cultivate V40‐large T immortalized and H‐ras transformed, WT and TAZ^KO^ Mouse Embryonal Fibroblasts at 37°C under a 5% CO_2_ humidified environment. The TAZ^KO^ cell line was generated as previously described (Ran *et al*, 2013) using CRISPR/Cas9 technology. Starvation conditions were simulated using Substrate limited media I (DMEM without glucose supplemented with 1 mM glutamine).

#### Control and patient‐specific iPSC‐CM

Ethics, the study was approved by the Ethics Committee of the University Medical Center Göttingen (approval number: 10/9/15) and carried out in accordance with the approved guidelines. Written informed consent was obtained from all participants before participation in the study. The experiments conformed to the principles set out in the WMA Declaration of Helsinki and the Department of Health and Human Services Belmont Report.

#### Generation and cardiac differentiation of iPSC lines

Human iPSC lines from a healthy donor and from a Barth syndrome patient with a pathological missense variant in *TAZ* were used in this study. Wild type iPSC line UMGi014‐C clone 14 (isWT1.14) was generated from dermal fibroblasts using the integration‐free Sendai virus and described previously (Rössler *et al*, 2021). The iPSC line UMGi054‐A clone 3 (iTAZ10.3) from a Barth syndrome patient carrying a heterozygous variant c.590G > T/p.Gly197Val in the *TAZ* gene was generated from dermal fibroblasts using the STEMCCA lentivirus system and described previously (Dudek *et al*, 2013, 2016). Human iPSC lines were differentiated into ventricular cardiomyocytes via WNT signaling modulation and subsequent metabolic selection and cultured for at least 30 days, as previously described (Kleinsorge & Cyganek, 2020). Human iPSCs and derived cardiomyocytes were cultured in feeder‐free and serum‐free culture conditions in a humidified incubator at 37°C and 5% CO_2_. Starvation conditions were simulated using Substrate limited media II (HBSS supplemented with 1 mM glutamine).

#### Cell compounds and treatments

Treatment with AMPK activator, under starvation conditions, for both MEF cells and iPSC cardiomyocytes was done with 10 nM of A‐769662 (AMPK activator) at the start of the starvation and in the duration of 8 h. The cells were further harvested for lysate preparation, were used for respirometry analysis or were subjected to an AMPK assay (all procedures described below).

### Mitochondrial isolation, PAGE analysis, and western blotting

#### Cells

Mitochondrial isolation from cells was done using previously described methods (Lazarou *et al*, 2009). Mechanical disruption using a Dounce homogenizer in TH buffer (10 mM KCl + 10 mM Hepes‐KOH (pH 7.4) + 300 mM Trehalose) was performed followed by differential centrifugation. Briefly, initial centrifugation at 400 *g* was done to remove debris and two consequent centrifugations at 10,000 *g* to pellet down and wash the mitochondria were performed.

#### Mouse cardiac tissue

Mitochondrial isolation from cardiac tissue was done as previously reported (Frezza *et al*, 2007). The heart was initially minced into small pieces and incubated in Digestion buffer (1× PBS + 10 mM EDTA + 0.05% Trypsin) for 30 min at room temperature. Following removal of the digestion buffer the tissue was subjected to mechanical disruption via homogenization using a Dounce homogenizer in Mitochondrial Isolation Buffer I (67 mM Sucrose + 50 mM Tris/Cl pH 7.4 + 50 mM KCl + 10 mM EDTA + 0.5% BSA; pH 7.4). The supernatant containing the mitochondria was separated from the tissue debris via a centrifugation at 700 *g*. The mitochondria were pelleted by a centrifugation at 8,000 *g* and finally washed using Mitochondrial Isolation Buffer II (250 mM Sucrose + 3 mM EGTA/Tris + 10 mM Tris/Cl pH 7.4; pH 7.4).

#### PAGE analysis, and western blotting

SDS–PAGE and gradient Tricine – SDS–PAGE (10–18%) were performed using standard methods. Tissue and cell lysis were done in RIPA buffer (25 mM Tris‐Cl pH 7.4 + 0.5% Na‐deoxycholate + 150 mM NACl + 0.1% SDS + 2 mM EDTA + 1% NP‐40+ 1× Protease Inhibitor Cocktail + 1× PhosphoSTOP). Protein concentration determination was done using a Bradford assay. Western blotting was done using standard semi‐dry transfer. Blue native PAGE analysis was performed using standard methods as previously described (Mick *et al*, 2012). Briefly, the solubilization of cardiac mitochondria to 1 mg/ml was done using a mitochondria solubilization buffer (40 mM Tris/Cl pH 7.4, 100 mM NaCl, 20% Glycerol, 0.2 mM EDTA pH 7.4) containing 1% digitonin for 30 min at 4°C. The samples were subjected to a 4–13% polyacrylamide gradient gel separation followed by a standard semi‐dry transfer.

### Proteomics and analysis of lipid profiles

#### Proteomics

##### Sample preparation for LC–MS/MS

Isolated mitochondria were lysed in 50 μl of lysis buffer (4% SDS, 1 mM EDTA, 100 mM Hepes pH 8, 2 × Protease Inhibitor Cocktail) and sonicated in Bioruptor (Diagenode) for 10 min using 15 s on/off cycles. Protein concentration in the lysate was determined using BCA assay kit (ThermoFisher Scientific) according to manufacturer's instructions. Twenty‐five micrograms total protein of each of the three WT and TAZ^G197V^ samples were reduced and alkylated following incubation with 10 mM tris(2‐carboxyethyl)phosphine (TCEP) and 40 mM 2‐chloroacetamide (CAA) for 30 min at 55°C. Protein clean‐up was performed according to the procedure by Hughes *et al* (2019). Proteins were digested with trypsin (MS grade, Promega) using 1:20 (wt/wt) trypsin‐to‐protein ratio and incubated overnight at 37°C in 100 mM TEAB buffer. Peptides were dried in SpeedVac (Eppendorf) and then re‐dissolved in 10% acetonitrile (ACN) 100 mM TEAB. Labeling reaction with respective TMT6plex labeling reagent (ThermoFisher Scientific) was conducted according to manufacturer's instructions. Labeled samples were pooled, dried in SpeedVac, and desalted using pre‐packed C18‐columns (Harvard apparatus). Desalted peptides were dissolved in 4% (v/v) ACN, 10 mM NH_4_OH pH 10 and fractionated using reversed‐phase chromatography under basic pH (bRP, buffer A: 10 mM NH_4_OH in water, pH ~ 10; buffer B: 10 mM NH_4_OH and 80% (v/v) ACN in water, pH ~ 10). For the bRP set‐up please refer to Silbern *et al*, 2021. Following the equilibration with 95% buffer A and 5% buffer B mixture, peptides were separated using a linear gradient ranging from 5 to 50% buffer B for 74 min followed by a washing step at 90% buffer B for 5 min. One‐minute fractions were collected and concatenated into 20 final fractions as suggested by Wang *et al*, 2011, and dried in SpeedVac.

##### LC–MS/MS

Dried peptides were re‐dissolved in 2% (v/v) ACN 0.1% (v/v) TFA in water and injected onto a C18 PepMap100‐trapping column (0.3 × 5 mm, 5 μm, Thermo Fisher Scientific) connected to an in‐house packed C18 analytical column (75 μm × 300 mm; Reprosil‐Pur 120 C18 AQ, 1.9 μm, Dr. Maisch GmbH). The columns were pre‐equilibrated using a mixture of 95% buffer A (0.1% (v/v) formic acid in water), 5% buffer B (80% (v/v) ACN, 0.1% (v/v) FA in water). Liquid chromatography was operated by UltiMate 3000 RSLC nanosystem (Thermo Fisher Scientific). Peptides were eluted using a 118 min‐linear gradient ranging from (i) 5 to 10% buffer B over 4 min, (ii) 10 to 38% buffer B over 72 min, (iii) 38 to 60% over 30 min, and ending with a washing step at 90% of buffer B for 5 min and a re‐equilibration step at 5% buffer B for 5 min. MS spectra were collected using an Orbitrap Fusion Tribrid mass spectrometer (Thermo Fisher Scientific). MS1 scans (350–1,650 *m/z*) were acquired in a positive ion mode with a resolution of 120,000 at 200 *m/z*, 5e5 automatic gain control (AGC) target, and 50 ms maximum injection time. Precursor ions (allowed charge states 2–7, dynamic exclusion 40 s) were isolated using a 1.6 *m/z* isolation window and fragmented at normalized collision energy (NCE) of 35%. MS2 fragment spectra were acquired with a resolution of 15,000, 2.5e5 AGC target, and 54 ms maximum injection time. Ten most intense fragment ions were selected for a subsequent SPS‐MS3 scan using an isolation window of 2 *m/z* and NCE of 55%. The SPS‐MS3 spectra were acquired at a resolution of 30,000 and maximum injection time of 54 ms.

##### LC–MS/MS data analysis

Raw files were processed using MaxQuant version 1.6.17.0 (Cox *et al*, 2011; Tyanova *et al*, 2016) using default settings except selection of reporter ions in MS3 (TMT6plex) for quantification. Canonical amino acid sequences of *mus musculus* proteins were retrieved from Uniprot (December 2021, 17,090 entries). Differential expression analysis was conducted in R statistical programming language using in‐house written scripts available upon request. In brief, matches to reverse sequences and potential contaminants reported by MaxQuant, as well as protein groups with less than two razor or unique peptides identified or protein groups containing four or more missing quantitative values were excluded from the analysis. Missing values were imputed for each TMT‐channel individually by random sampling from a gauss distribution with a mean at the 5% quantile and a half standard deviation of the log‐transformed intensities. Log_2_‐transformed reporter ion intensities were then normalized using Tukey median polishing and subjected to statistical testing using limma package (Smyth, 2005). Differences in protein group intensities between PD‐ and control samples were expressed as log_2_ fold changes (Mutant/WT). Protein groups showing Empirical‐Bayes moderated *P*‐values were corrected for multiple testing using the *q*‐value approach. Protein groups with *q*‐value < 0.01 and absolute log_2_ fold change > log_2_ (1.5) were considered as differentially expressed and subjected for further bioinformatic analysis.

##### Analysis of lipid profiles by mass spectrometry

Mitochondria were subjected to lipid extractions using an acidic liquid–liquid extraction method (Paltauf & Hermetter, 1994; Özbalci *et al*, 2013). In order to ensure that similar amounts of lipids were subjected to extractions, a test extraction was performed to determine the concentration of phosphatidylcholine (PC) as a bulk membrane lipid and to adapt extractions volumes to similar total lipid amounts. Test extractions were done in the presence of 25 pmol phosphatidylcholine PC standard mix (PC 13:0/13:0, 14:0/14:0, 20:0/20:0; 21:0/21:0, Avanti Polar Lipids). The final chloroform phase of the liquid–liquid extraction was evaporated under a gentle stream of nitrogen at 37°C. Samples were either directly subjected to mass spectrometric analysis, or were stored at −20°C prior to analysis, which was typically done within 1–2 days after extraction. Lipid extracts were resuspended in 10 mM ammonium acetate in 60 μl methanol. Two μl aliquots of the resuspended lipids were diluted 1:10 in 10 mM ammonium acetate in methanol in 96‐well plates (Eppendorf twin tec 96) prior to measurement. Samples were analyzed on an QTRAP 6500+ mass spectrometer (Sciex) with chip‐based (HD‐D ESI Chip, Advion Biosciences) electrospray infusion and ionization via a Triversa Nanomate (Advion Biosciences). MS settings and scan procedures are listed in Dataset EV1. Data evaluation was done using LipidView (Sciex) and an in‐house‐developed software (ShinyLipids). For the quantification of CL and MLCL species, sample volumes corresponding to an average PC amount of approximately 650 pmol was subjected to extractions. Quantification was achieved by adding the following internal lipid standards: 25 pmol MLCL (16:0/16:/16:0, Avanti Polar Lipids), and 50 pmol of a Cardiolipin mix containing CL 57:4 (14:1(3)‐15:1), CL 61:1 (15:0(3)‐16:1), CL 80:4 (22:1(3)‐14:1) and CL86:4 (24:1(3)‐14:1; Cardolipin mix I, Avanti Polar Lipids). Following extraction and evaporation, 5 μl aliquots of the reconstituted lipid extracts were diluted with 15 μl 7.5 mM ammonium formate in isopropanol:methanol:chloroform (v:v:v, 4:2:1). Samples were subjected to MS analysis on a Q Exactive Thermo Fisher Scientific using a chip‐based (HD‐D ESI Chip, Advion Biosciences) electrospray infusion and ionization via a Triversa Nanomate (Advion Biosciences). MS settings and scan procedures are listed in Dataset EV1.

Lipid species evaluation was done using the open‐source tool LipidXplorer 1.2.8.1 (https://zenodo.org/record/3570469; Herzog *et al*, 2011, 2012) and PeakStrainer (Schuhmann *et al*, 2017) for Q Exactive data, LipidView (Sciex), for QTRAP 6500+ data and in‐house‐developed tools (ShinyLipids and ShinyLipidcountr_alpha4). The amount for endogenous molecular lipid species was calculated based on the intensities of the internal standards.

### RNA Isolation, cDNA synthesis, and qPCR

RNA isolation from cells and tissues was done using TRIzol™ reagent. The isolation was performed as instructed by the manufacturer (Thermo Fisher Scientific). The isolated RNA was used for reverse transcription into cDNA or subjected to RNA sequencing. Using RevertAid First Strand cDNA Synthesis Kit (Thermo Fisher Scientific), isolated RNA was transcribed using random hexamer primers to generate cDNA. The cDNA was subjected to qPCR analysis using the SensiMix™ SYBR® Low‐ROX kit (Meridian Bioscience). The cDNA from each sample was pipetted in triplicate and the entire reaction was performed in a Quant studio 6 flex Cycler. Primer sequences used for qPCR are available upon request.

### RNA sequencing

cDNA libraries were prepared with 500 ng of total RNA using the TrueSeq® Library preparation kit (Illumina, USA). Quality control for libraries was done using the Bioanalyzer DNA 1000 (Agilent Technologies, USA), and dsDNA concentration was measured with Qubit 2.0 Fluorometer (Life Technologies, USA). The HiSeq 2000 sequencing platform (Illumina, USA) was used to perform 50 bp single‐end sequencing on the samples.

#### Data processing and bioinformatic analyses

For processing of the sequencing data, a customized in‐house software pipeline was used. Illumina's bcl2fastq (v2.20.0) was used to convert the base calls in the per‐cycle BCL files to the per‐read FASTQ format from raw images. Along with base calling, adaptor trimming, and demultiplexing were performed. Quality control of raw sequencing data was performed using FastQC (v 0.11.9). Reads were then mapped to the genome (mm10 for mouse data and hg38 for human iPSC data) using the splice‐aware aligner STAR (v2.7.3a; Dobin *et al*, 2013), with a maximum number of two mismatches allowed per pair, and other default parameters. The number of aligned reads overlapping with exons for each gene was then counted with the featureCounts program from the Subread package (v2.0.0; Liao *et al*, 2013, 2014). To obtain differentially expressed genes, we first identified unwanted sources of variation and corrected them using the svaseq function from the sva R package (v3.40.0; Leek *et al*, 2012). We then used the DESeq2 R package (v1.32.0; Love *et al*, 2014) to perform the differential expression analysis. Genes that passed a cut‐off of baseMean ≥ 50, fold change > 1.15 or < −1.15, and adjusted *P*‐value < 0.05 were considered to be significantly differentially expressed. Over‐representation (pathway enrichment) analysis of the up and downregulated genes was done using the WebGestaltR R package (version 0.4.4; Liao *et al*, 2019) with KEGG database terms (Kanehisa & Goto, 2000). Plots were created in R (version 4.1.0) using custom scripts. ClueGO (v2.5.8; Bindea *et al*, 2009) and Cytoscape (v3.9.1; Shannon *et al*, 2003) were used to build customized networks and visualizations of the deregulated genes/pathways.

### Real time respirometry

#### Mito stress test

For the determination of the Oxygen Consumption rate (OCR) in cells as well as isolated mitochondria a XF96e Extracellular Flux Analyzer was used. The assay was performed as described by the manufacturer in Seahorse XF Cell Mito Stress Test Kit User Guide (103016‐400, Agilent Technologies). Fifty thousand cells were plated on a Seahorse Plate for this assay. Baseline respiration was measured in XF DMEM buffer (supplemented with 1 mM pyruvate, 2 mM glutamine, and 10 mM glucose) for MEF cells and XF RPMI buffer (supplemented with 1 mM pyruvate, 2 mM glutamine, and 10 mM glucose) for iPSC cardiomyocytes. Oxygen consumption was further measured in differing metabolic conditions following the addition of 3 μM Oligomycin, 1.5 μM CCCP and 0.5 μM Antimycin/Rotenone.

Mitochondrial Assay Buffer (70 mM Sucrose, 210 mM Mannitol, 5 mM HEPES, 1 mM EGTA, 10 mM KH_2_PO_4_, 5 mM MgCl_2_ 0.5% BSA 40 mM ADP (added fresh)) supplemented with either 10 mM Pyruvate/4 mM Malate/4 mM ADP or 10 mM Succinate/4 mM ADP was used to measure basal respiration for isolated mitochondria.

#### Glycolysis stress test

Extracellular acidification rate of cells was measured using XF96e Extracellular Flux Analyzer. The assay was performed as specified in Seahorse XF Glycolysis Stress Test Kit User Guide (103020‐400, Agilent Technologies) by the manufacturer. Fifty thousand cells were plated on a Seahorse Plate for this assay. In short basal acidification of the media was measured in XF DMEM Buffer (supplemented with 1 mM pyruvate and 2 mM glutamine), followed by consequent measurements upon the addition of 10 mM Glucose 3 μM Oligomycin, and 50 mM 2‐deoxy‐D‐glucose.

#### Palmitate oxidation stress test

Seahorse XF Palmitate Oxidation Stress Test Kit was used to asses long chain fatty acid oxidation via the Oxygen consumption rate of living cells (MEFs and iPSC cardiomyocytes) on a XF96e Extracellular Flux Analyzer. The assay was performed as described in the XF Palmitate Oxidation Stress Test Kit: Advanced Assay user manual (103693‐100, Agilent Technologies) provided by the manufacturer. Shortly, 50,000 cells were plated and were left to adhere to the plate. The media was changed to Substrate limited media I for MEF cells and Substrate limited media II for iPSC cardiomyocytes and the plate was incubated overnight at 37°C. One hour before the measurement the media was changed to either XF DMEM or XF RPMI (supplemented with 0.5 mM Glucose, 1 mM Glutamine, 1% FBS and 0.5 mM Carnitine). Ten minutes before the measurement 4 μM ETO was added to the negative control wells. Right before the measurement Palmitate/BSA was added to the wells. Oxygen consumption measured as basal and in differing metabolic conditions following the addition of 3 μM Oligomycin, 4 μM CCCP and 0.5 μM Antimycin/Rotenone. For the purposes of this experiment the data was normalized using CyQuant™ Direct cell proliferation assay (as instructed by the manufacturer, Thermo Fisher Scientific) to 75,000 cells per well.

For the purpose of measuring long chain fatty acid oxidation in isolated mitochondria from mouse heart a XF96e Extracellular Flux Analyzer was used. The mitochondria were freshly isolated at 4°C as described above and plated in a Seahorse plate resuspended in Respiration Buffer (214 mM Sucrose, 12.8 mM KCl, 0.86 mM EGTA, 4.3 mM MgCl_2_, 25.7 mM K_2_HPO_4_ 80 μM Palmitoyl, 4.3 μM Carnitine, 45 mM Malate, 3.7 mM ADP; ph 7.4) at room temperature. Addition of 0.2 mM ETO in the buffer was used as negative control. The basal Oxygen consumption rate was measured. For the measurement of oxygen consumption rate of mitochondria isolated from frozen heart samples the protocols (Basic Protocol 1) described in Osto *et al*, 2020 was followed to isolate the mitochondria, followed by the steps described above for the measurement.

#### Real‐time ATP production rate

Quantification of the real‐time production rate of ATP, produced both by mitochondrial respiration and glycolysis was done using XF96e Extracellular Flux Analyzer. The assay and subsequent quantification were done as described in the Agilent Seahorse XF Real‐Time ATP Rate Assay Kit user guide (103592‐400 Rev B0, Agilent Technologies) provided by the manufacturer. In short both Oxygen consumption rate and Extracellular acidification rate were measured as basal as well as upon the subsequent addition of 3 μM Oligomycin and 1.5 μM antimycin/rotenone. To quantify the ATP production rate Agilent Seahorse XF Real‐Time ATP Rate Assay Report Generator (S7888‐10012, Agilent Technologies) was used.

### Complex activity assays

Complex IV Rodent Enzyme Activity Microplate Assay Kit (ab109911), Complex I Enzyme Activity Microplate Assay Kit (Colorimetric; ab109721) and Complex II Enzyme Activity Microplate Assay Kit (ab109908) were used to perform complex IV, complex I and complex II activity assays respectively using isolated mitochondria from the mouse heart. The assays were performed following the instructions provided by the manufacturer (Abcam) in ELIZA based plate assays. Absorbance at 550 nm was measured to determine complex IV activity. Absorbance at 450 nm was measured to determine complex I activity. Absorbance at 600 nm was measured to determine complex II activity. The measurements were performed on a Synergy H1 microplate reader (BioTek).

### AMPK assay

In order to detect phosphorylated AMPK in cellular samples upon starvation EnzyFluo™ AMPK Phosphorylation Assay Kit (EAMPK‐100) was used. The assay was performed as indicated in the instruction manual from the manufacturer (Universal Biologicals). The final output of the assay was measured on a Synergy H1 microplate reader (BioTek).

### ATP assay (luminescent)

Total cellular ATP was measured using the Luminescent ATP Detection Assay Kit (ab113849, Abcam). The assay was performed as per the manufacturers' instructions and the final output was measured on a Synergy H1 microplate reader (BioTek).

### Lactate assay

L‐Lactate Assay Kit (Colorimetric; ab65331, Abcam) was used to detect L(+)‐Lactate in mouse heart tissue lysate. The assay was performed as described by the manufacturer. The final output was measured at OD450 on a Synergy H1 microplate reader (BioTek).

### ROS measurement

To measure mitochondrial superoxide anion production, MitoSOX™ Red was used for staining isolated cardiac mitochondria at a concentration of 3 μM, following the protocol provided by the manufacturer (Invitrogen). Using BD‐Canto flow cytometer (Becton Dickinson), 10,000 gated events were recorded per sample and analyzed using the FACS‐Diva software in order to obtain the final result.

### Echocardiography

Echocardiography was performed to evaluate the heart function of the animals at age 8‐, 14‐ and 20‐weeks. The animals were anesthetized with isoflurane (initial anesthesia was performed in the induction chamber with mixture of 4–5% isoflurane per 1 l of oxygen), placed to the pre‐warm stage and fixed with the tape to the stage. Animals were kept anesthetized by switching to inhalation mask through the nasal mask (mixture of 1–3% isoflurane and oxygen with flow 0.3–0.5 l/min). The chest of animals was shaved by shaving gel and the pre‐warmed sonographic gel was applied to the chest. Ultrasound probe (MS400 transducer, VisualSonic, FujiFilm) connected to the machine (Vevo2100, VisualSonic, Fujifilm) was used to record cardiac function in parasternal short (SAX) and long axis. Images in B‐mode and M‐mode were obtained and analyzed using by Auto LV function, as well as strain analysis was performed (VevoLab, Fujifilm). The data were exported to Microsoft Excel, and analyzed in GraphPrism software. Performed analysis 2‐way ANOVA with Šídák's multiple comparisons test, *P* levels < 0.05*, < 0.01**, < 0.0001****.

### AMPK activator treatment

The basal echocardiography confirming the development of cardiac dysfunction in TAZ^G197V^ mice was performed at the age of animals 16–17 weeks, and after it, the treatment of animals with AMPK activator (A‐769662, MedChemExpress, cat. No. HY‐50662) had started. The animals were divided into four experimental groups – wild type mice with vehicle administration (WT + veh, *n* = 15), WT with AMPK activator administration (WT + AMPK, *n* = 9) TAZ^G197V^ with vehicle administration (TAZ + veh, *n* = 4) and TAZ^G197V^ animals with administration of AMPK activator (TAZ + AMPK, *n* = 7). The AMPK activator (30 mg/kg) was dissolved in vehicle (ratio: 40% peg400, 5% tween80, 55% physiological solution 0.9% NaCl). AMPK activator or vehicle were administrated i.p. to each group once daily for 6 weeks. Echocardiography and ECG were recorded every 2 weeks after the initiation of treatment (2 weeks, 4 weeks and final 6 weeks). Mice were housed in a pathogen free animal environment with 12 h light/12 h dark cycles under controlled temperature and food *ad libidum*. The health check and food intake were performed every day and body weight was recorded. All procedures were performed in accordance with the regulations of the Institutional Animal Care and Use Committee of the Institute of Molecular Genetics, Czech Academy of Sciences (Prague, Czech republic).

### Histology

Hearts were fixed in 4% paraformaldehyde. Tissues were processed on Leica ASP6025 automatic vacuum tissue processor and embedded in Leica EG1150 H + C embedding station. For sectioning, Leica RM2255 rotary microtome (2 μm sections) was used. Sections were deparaffined and hydrated in xylene and descending ethanol solutions and subsequently stained by Picrosirius red (Direct red 80, Sigma Aldrich; 0.5 g picrosirius powder stain/500 ml picric acid solution). The nuclei were counterstained by Weigert's hematoxylin kit (Sigma Aldrich) and followed by manufacture instructions. Slides were dehydrated in and mounted with coverslips in automatic staining system (Leica ST5010‐CV5030). Stained slides were digitalized in slide scanner AxioScan Z.1 (Zeiss).

### Quantification and statistics

The calculation of Standard Error mean (SEM) as well as the significance in difference between groups was performed using Prism 9 for macOS software (GraphPad Software, San Diego, CA) unless specifically stated otherwise. The tests used were either unpaired non‐parametric or paired non‐parametric *t*‐tests and ANOVA, unless otherwise noted; *P* levels < 0.05*, < 0.01**, < 0.001***, < 0.0001****. All replicates labeled as “*n*=” are biological replicates for their respective experiments.

### Study design

Sample size was chosen based on previous experience with similar biochemical analysis. Data evaluation and processing was done by different scientists. No blinding was done in the analysis.

## Author contributions

**Peter Rehling:** Conceptualization; resources; supervision; funding acquisition; writing – original draft; project administration; writing – review and editing. **Arpita Chowdhury:** Conceptualization; data curation; formal analysis; supervision; investigation; writing – original draft; writing – review and editing. **Angela Boshnakovska:** Conceptualization; data curation; software; formal analysis; supervision; validation; investigation; visualization; writing – original draft; writing – review and editing. **Abhishek Aich:** Formal analysis; investigation; writing – original draft. **Aditi Methi:** Data curation; software; visualization; writing – original draft. **Ana Maria Vergel Leon:** Data curation; formal analysis; writing – original draft. **Ivan Silbern:** Data curation; software; writing – original draft. **Christian Lüchtenborg:** Data curation; software; formal analysis. **Lukas Cyganek:** Resources; methodology; writing – original draft. **Jan Prochazka:** Resources; methodology; writing – original draft. **Radislav Sedlacek:** Formal analysis. **Jiri Lindovsky:** Formal analysis. **Dominic Wachs:** Formal analysis. **Zuzana Nichtova:** Software; formal analysis; writing – original draft. **Dagmar Zudova:** Formal analysis. **Gizela Koubkova:** Formal analysis; project administration. **André Fischer:** Conceptualization; supervision; funding acquisition; writing – original draft. **Henning Urlaub:** Supervision; funding acquisition; methodology; writing – original draft. **Britta Brügger:** Supervision; funding acquisition; methodology; writing – original draft. **Dörthe M Katschinski:** Conceptualization; supervision; funding acquisition; writing – original draft; writing – review and editing. **Jan Dudek:** Formal analysis; writing – original draft; writing – review and editing.

## Disclosure and competing interests statement

A. Chowdhury is now an employee of Dewpoint Therapeutics GmbH. Christian Lüchtenborg is now an employee of BioNTech. The authors declare no competing financial interests.

## For more information

This space should be used to list relevant web links for further consultation by our readers. Could you identify some relevant ones and provide such information as well? Some examples are patient associations, relevant databases, OMIM/proteins/genes links, author's websites, etc…

- Lab website: https://biochemie.uni‐goettingen.de/index.php/mitochondrial‐protein‐biogenesis‐v‐2/
- OMIM website: https://www.omim.org/entry/300394 TAZ gene; https://www.omim.org/entry/302060 Barth syndrome
- Barth syndrome foundation website: https://www.barthsyndrome.org/

## Supporting information



Expanded View Figures PDF

Dataset EV1

Dataset EV2

PDF+

Source Data for Figure 1

Source Data for Figure 2

Source Data for Figure 4

Source Data for Figure 5

Source Data for Figure 6

Source Data for Figure 7

## Data Availability

The datasets produced in this study are available in the following databases:
RNA‐Seq (Mouse, Human): Gene Expression Omnibus (GEO) DataSets – SuperSeries (accession: GSE221664) [URL: https://www.ncbi.nlm.nih.gov/geo/query/acc.cgi?acc=GSE221664]. RNA‐Seq (Mouse): Gene Expression Omnibus (GEO) DataSet – SubSeries (accession: GSE221662) [URL: https://www.ncbi.nlm.nih.gov/geo/query/acc.cgi?acc=GSE221662]. RNA‐Seq (Human): Gene Expression Omnibus (GEO) DataSet – SubSeries (accession: GSE221663) [URL: https://www.ncbi.nlm.nih.gov/geo/query/acc.cgi?acc=GSE221663]. Mass Spectrometry (Mouse): MassIVE DataSet (accession: MSV000092116) [URL: https://massive.ucsd.edu/ProteoSAFe/dataset.jsp?task=3896a486c8934743b250a95d491cabc9].
