# Supplementary material for: Metabolic switch from fatty acid oxidation to glycolysis in knock‐in mouse model of Barth syndrome
Source: EMBO Mol Med. 2023 Aug 3;15(9):e17399. doi: 10.15252/emmm.202317399 (PMC10493589; doi:10.15252/emmm.202317399)
Supplement: Supplementary file 1 — Expanded View Figures PDF [file EMMM-15-e17399-s004.pdf]

# Expanded View Figures

**Figure EV1. Characterization of the TAZ$^{G197V}$ mouse model.**

A Presentation of Crispr/Cas9 generated mutation in Exon 7 in the gene encoding Tafazzin.
B Western blot analysis of steady state protein levels from isolated heart mitochondria of 20-week-old female TAZ$^{G197V}$ heterozygous mice.
C Body weight of female WT and TAZ$^{G197V}$ heterozygous mice. Mean ± SEM, $n = 8$, 2Way ANOVA Multiple comparisons: *$P < 0.05$.
D Steady state protein levels analyzed by western blotting of isolated mitochondria from brain, liver, and muscle of 12-week-old male mice.
E–J Quantification of stroke volume (E), cardiac output from parasternal short axis (SAX) (F), left ventricle posterior systolic wall thickness analyzed from parasternal short axis (SAX) (G), Radial systolic strain (H) Circumferential systolic strain (I), And longitudinal diastolic strain (J). Mean ± SEM, $n = 9$, 2Way ANOVA Multiple comparisons: *$P < 0.05$, **$P < 0.01$, ****$P < 0.0001$.

 © 2023 The Authors

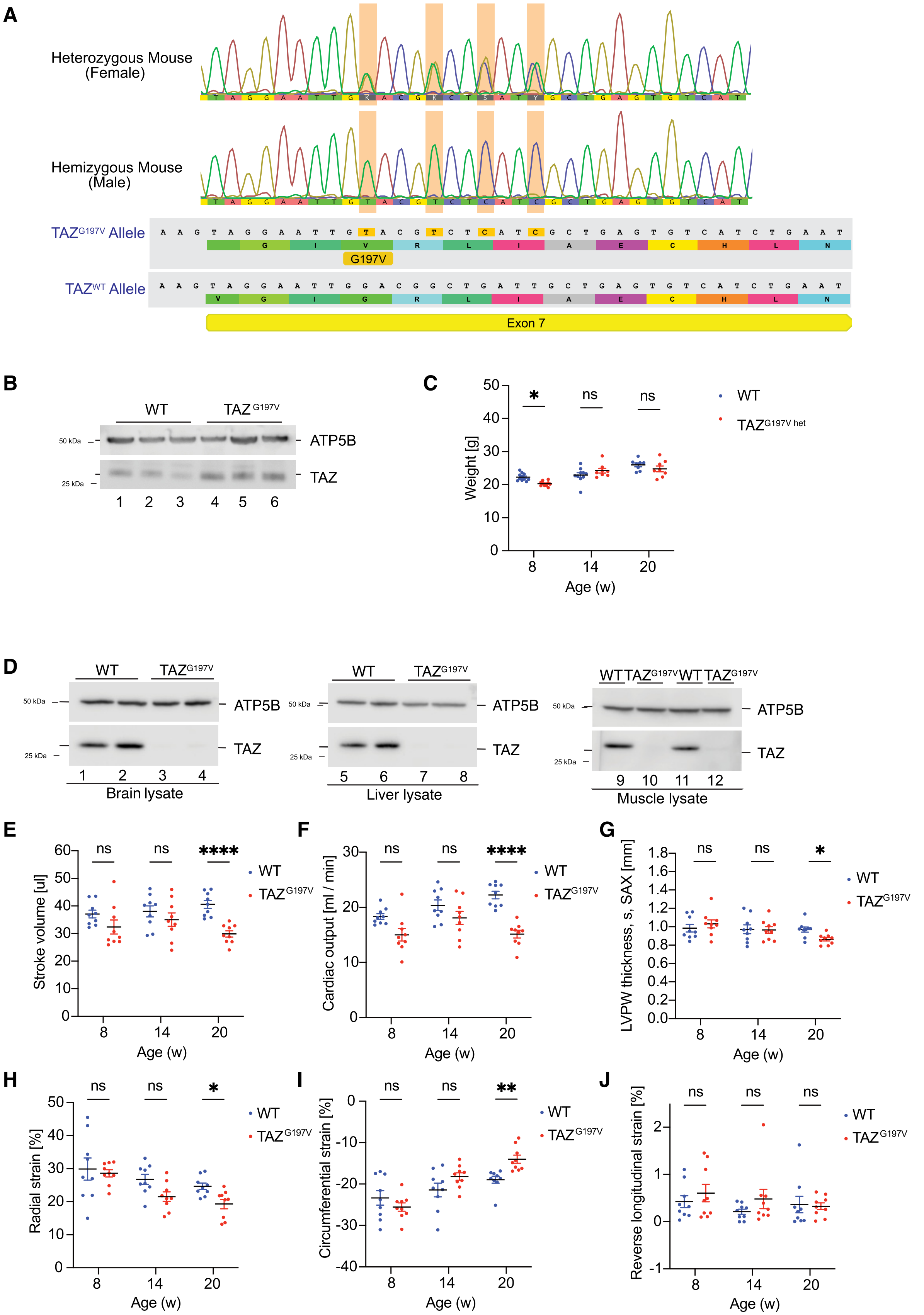

A
Heterozygous Mouse (Female)
Hemizygous Mouse (Male)
TAZG197V Allele
G197V
TAZWT Allele
Exon 7
B
WT
TAZ G197V
ATP5B
TAZ
50 kDa
25 kDa
1 2 3 4 5 6
C
Weight [g]
Age (w)
WT
TAZG197V het
D
Brain lysate
Liver lysate
Muscle lysate
E
Stroke volume [ul]
F
Cardiac output [ml / min]
G
LVPW thickness, s, SAX [mm]
H
Radial strain [%]
I
Circumferential strain [%]
J
Reverse longitudinal strain [%]


**Figure EV1.**

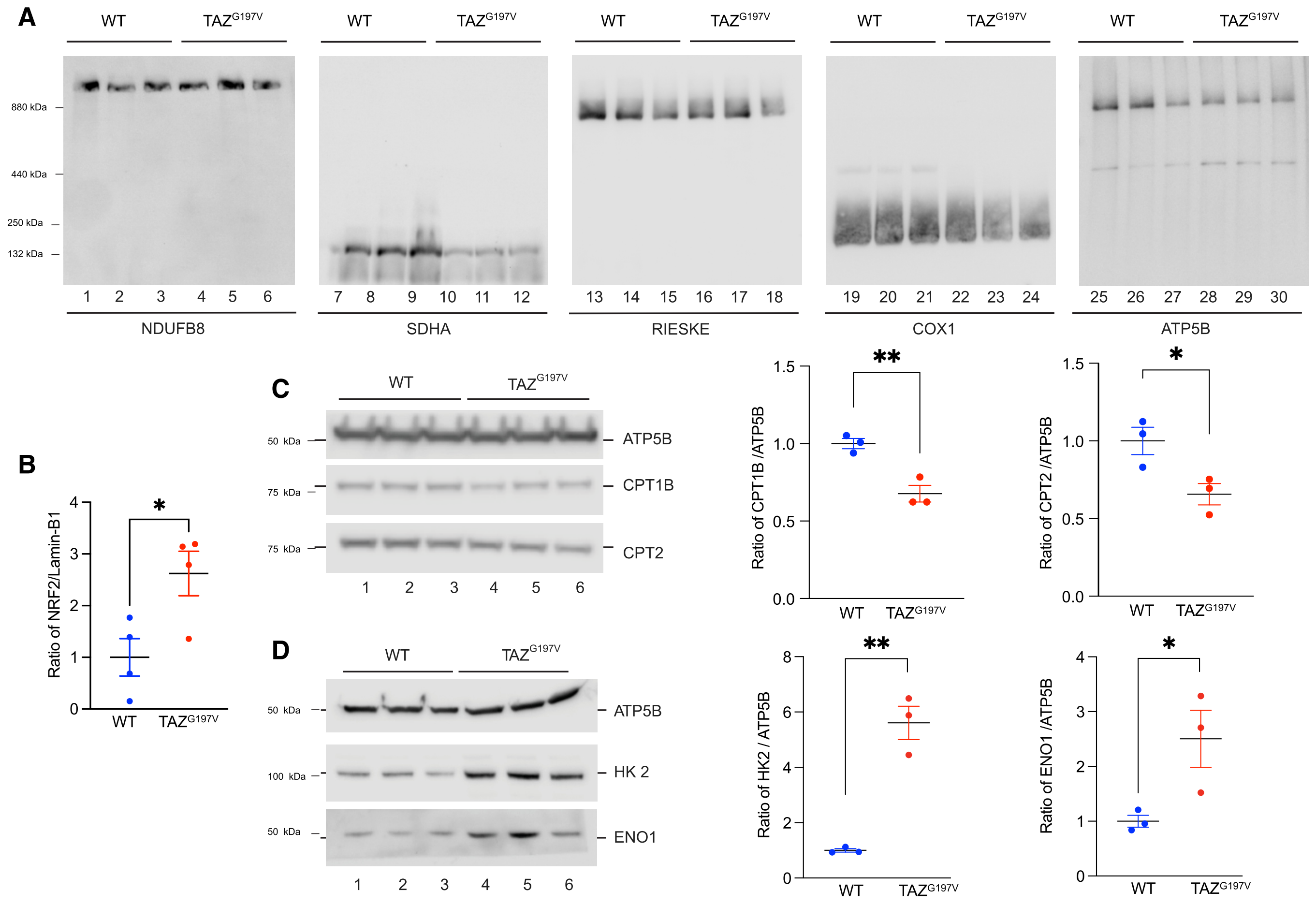


**Figure EV2. Molecular analysis of the TAZ$^{G197V}$ mouse.**

A BN-PAGE analysis of heart mitochondria solubilized in 1% DDM (12-week-old mice).

B Quantification of NRF2 levels on Western blot normalized to Lamin-B1. Mean ± SEM, $n$ = 4, unpaired $t$-test: *$P$ < 0.05.

C Western blot analysis of isolated mouse heart (20-week-old mice) mitochondria (left) and quantification (right) of CPT1 and CPT2 protein levels normalized to ATP5B. Mean ± SEM, $n$ = 3, unpaired $t$-test: *$P$ < 0.05, **$P$ < 0.01.

D Western blot analysis as in a (left) and quantification of HK2 and ENO1 normalized to ATP5B. Mean ± SEM, $n$ = 3, unpaired $t$-test: *$P$ < 0.05, **$P$ < 0.01.


© 2023 The Authors

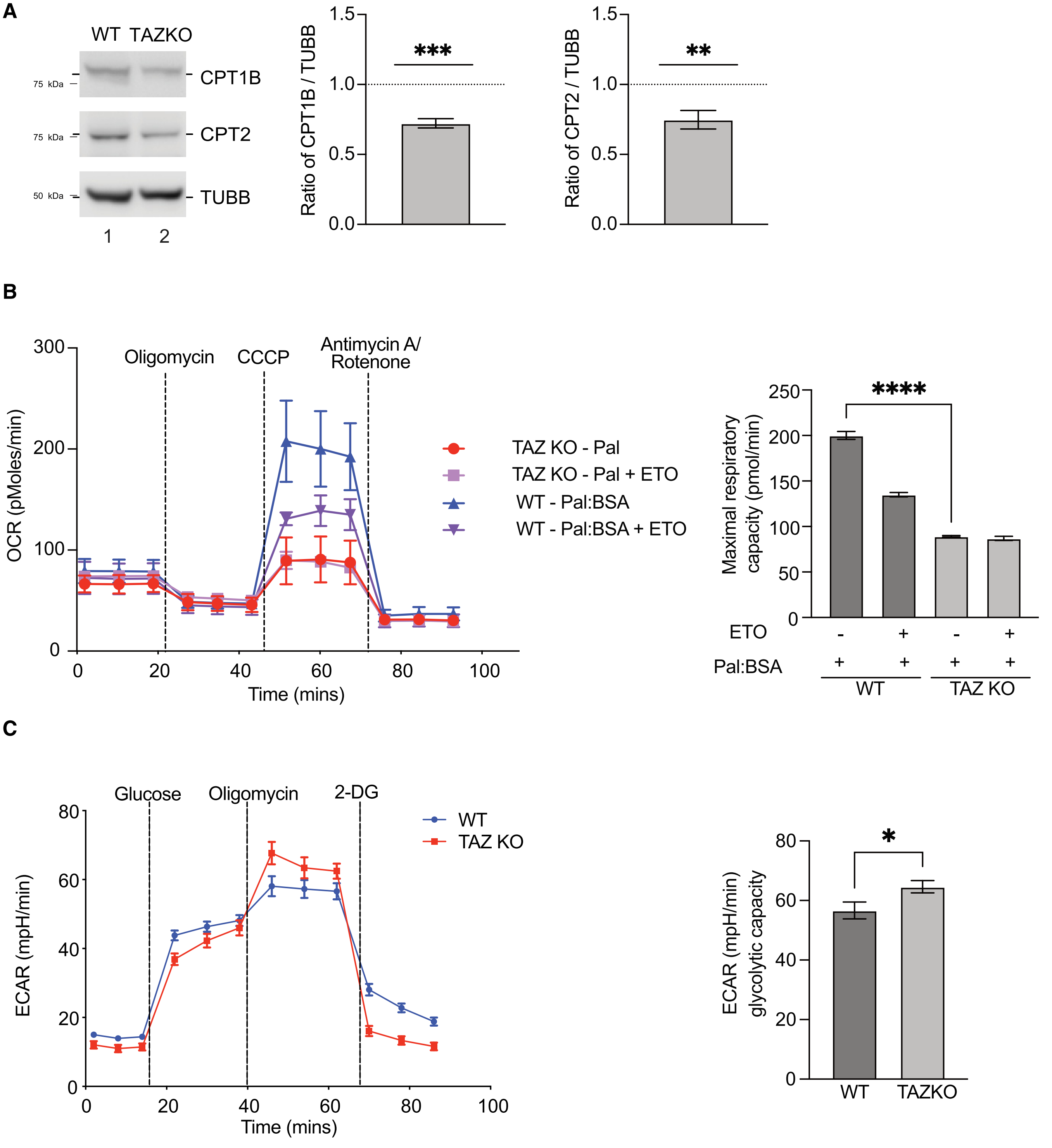


**Figure EV3. TAZ-deficient MEF cells display reduced fatty acid oxidation.**

A Western blot analysis (left) and quantification (right) of CPT1 and CPT2 protein levels in WT and TAZKO MEF cell lysate normalized to β-tubulin. Mean ± SEM, $n = 4$, unpaired $t$-test: $**P < 0.01$, $***P < 0.001$.

B Oxygen consumption rate of WT and TAZ MEF cells in media supplemented with palmitate-BSA, basal, oligomycin, CCCP, and antimycin/rotenone treated (left). ETO, etomoxir. Quantification of maximal respiratory capacity (right). Mean ± SEM, $n = 3$, unpaired $t$-test: $****P < 0.0001$.

C Extracellular acidification rate, presentation of basal rate, addition of glucose, oligomycin, and 2 deoxyglucose (2-DG). Quantification of Glycolysis and Glycolytic capacity of the cells is presented on the right. Mean ± SEM, $n = 6$, unpaired $t$-test: $*P < 0.05$.

© 2023 The Authors

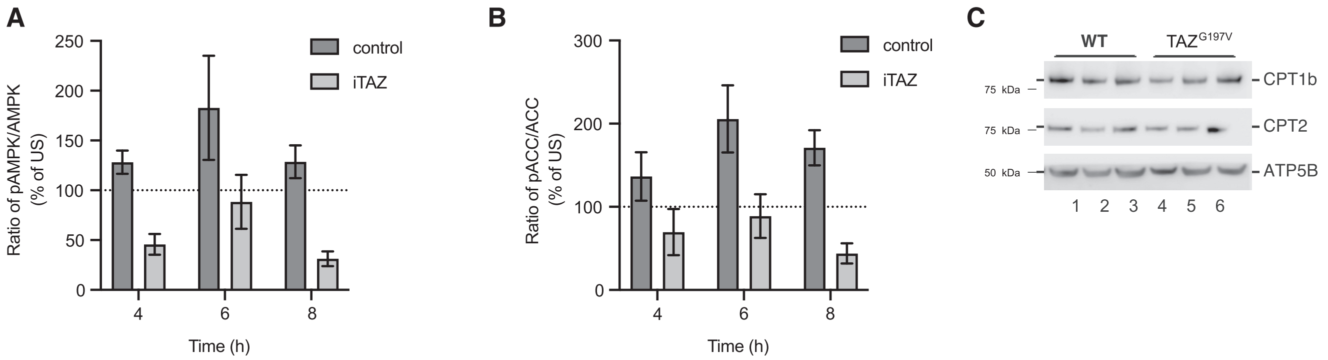


**Figure EV4. Altered AMPK signaling in iPSC-cardiomyocytes and mice.**

A Quantification of pAMPK/AMPK and.

B pACC/ACC ratio, under starvation conditions at indicated hours for control and iTAZ iPSC-cardiomyocytes. Unstarved ratio set as 100%. Mean ± SEM, *n* = 3.

C CPT1 and CPT2 protein levels from isolated heart mitochondria of 24-week-old mice. Mice were treated with A-769662 (AMPK activator) for 6 weeks prior to analysis.

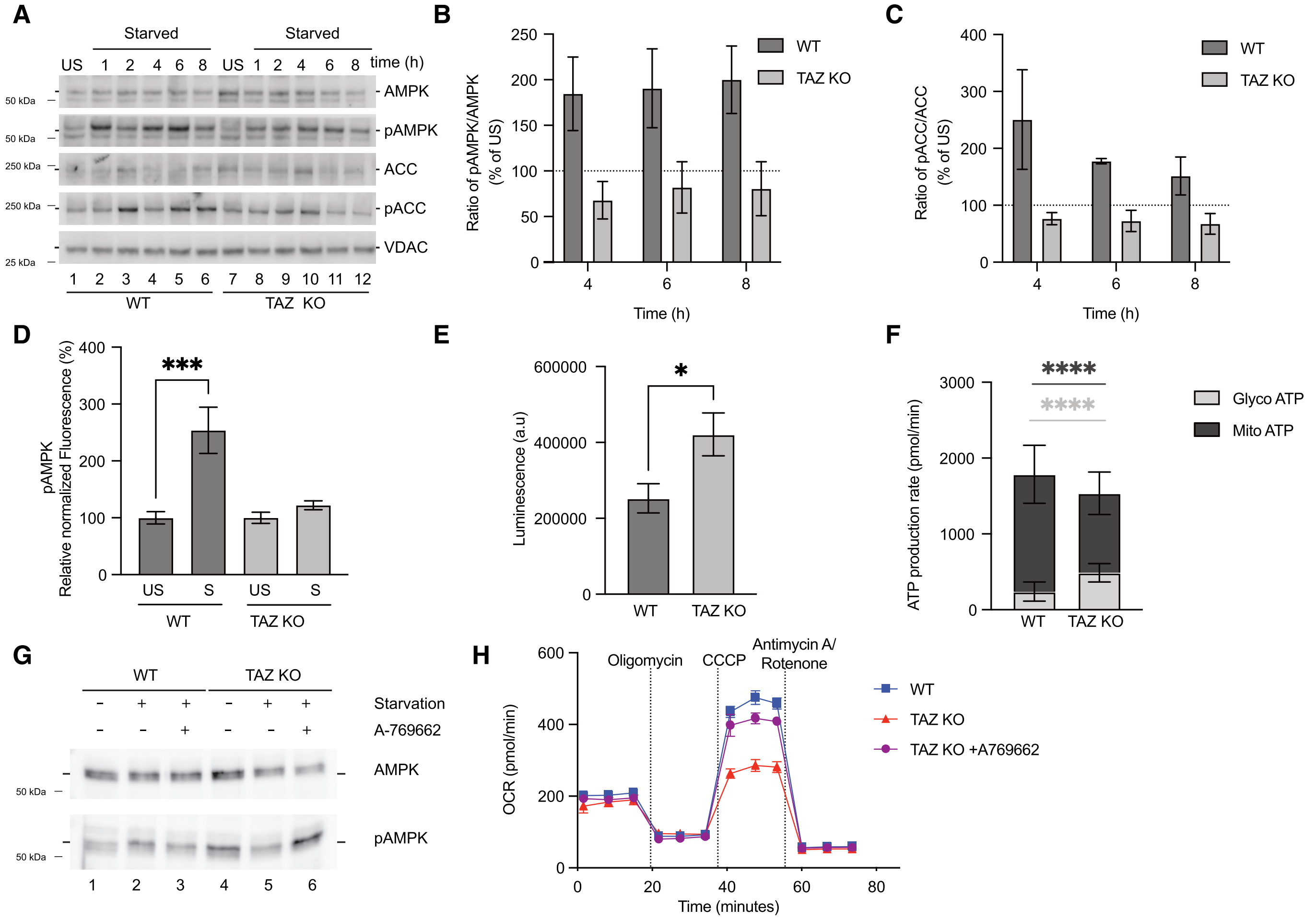


**Figure EV5.**

 © 2023 The Authors

◀ **Figure EV5. Altered AMPK signaling in MEF cells.**

A Western blot analysis of steady state protein levels of WT and TAZ KO MEF cell lysates. Cells were subjected to starvation for indicated times or left unstarved.

B, C Quantification of pAMPK/AMPK and (C) pACC/ACC ratio, under starvation conditions at indicated hours for WT and TAZ KO MEF cells. Unstarved ratio set as 100%. Mean ± SEM, $n = 3$.

D Amount of pAMPK in WT and TAZ KO MEF cell lysate. Cells were subjected to 8 h of starvation. Fluorescence signal was normalized to total protein amount. Mean ± SEM, $n = 3$, unpaired $t$-test: ***$P < 0.001$.

E Quantification of total ATP amounts (arbitrary luminescence units) in WT and TAZ KO MEF cell lysate. Mean ± SEM, $n = 3$, unpaired $t$-test: *$P < 0.05$.

F Real-time ATP rate assay to measure ATP production rate (glycolytic and mitochondrial). Mean ± SEM, $n = 3$, unpaired $t$-test: ****$P < 0.0001$.

G Western blot analysis of steady state protein levels of cell lysates from cells treated as indicated. A-769662 (AMPK activator).

H Real-time respirometry, basal oxygen consumption rate (OCR), oligomycin, CCCP and antimycin/rotenone treated for WT and TAZ KO (non-treated and A-769662 treated). Mean ± SEM, $n = 3$.

© 2023 The Authors
