# Supplementary figures and images for: Metabolic switch from fatty acid oxidation to glycolysis in knock‐in mouse model of Barth syndrome
Source: EMBO Mol Med. 2023 Aug 3;15(9):e17399. doi: 10.15252/emmm.202317399 (PMC10493589; doi:10.15252/emmm.202317399)

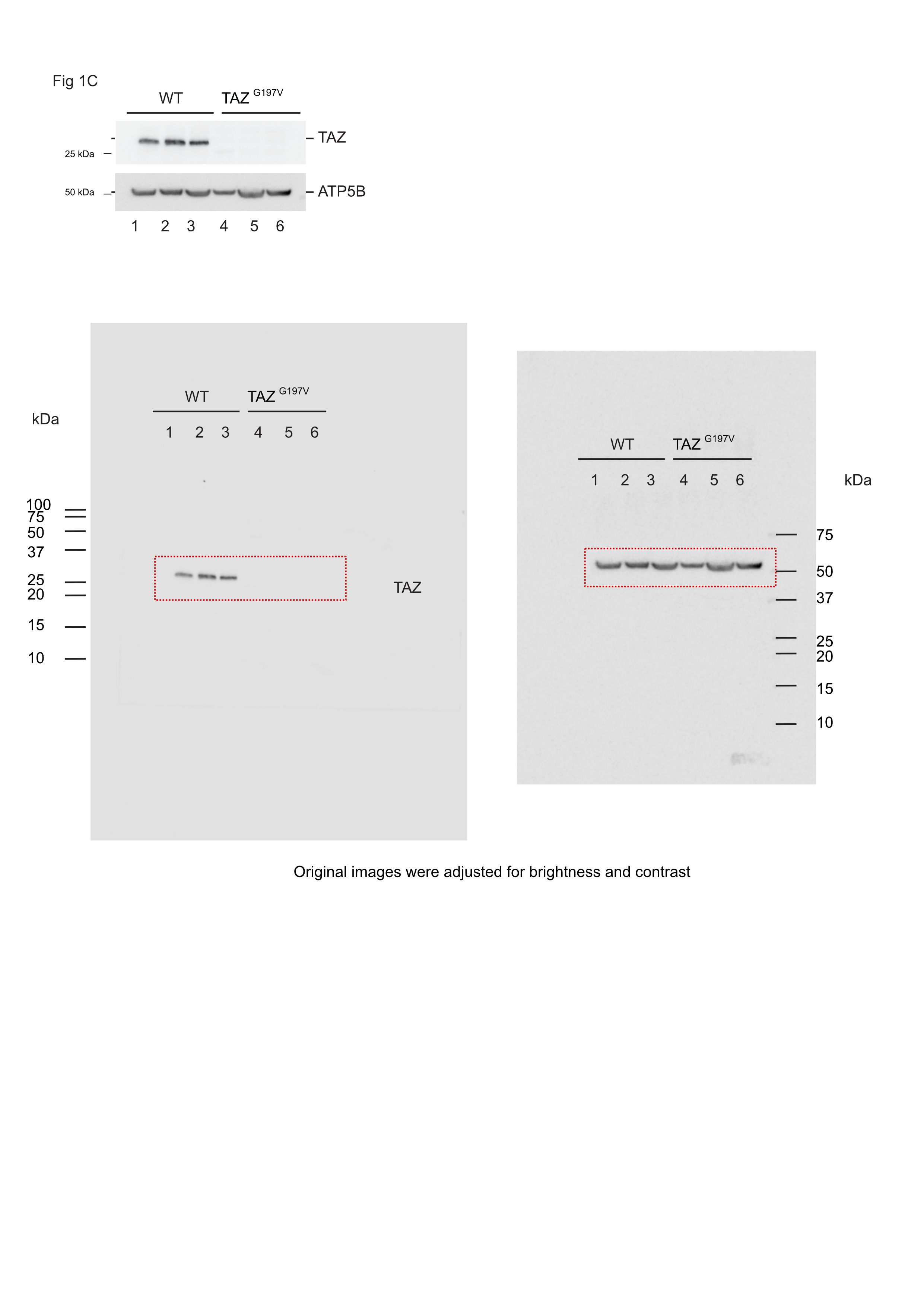

Supplement: Supplementary file 5 — Source Data for Figure 1 [file EMMM-15-e17399-s003.zip › Figure 1/1C/Tafazzin:ATP5B blot.tiff]

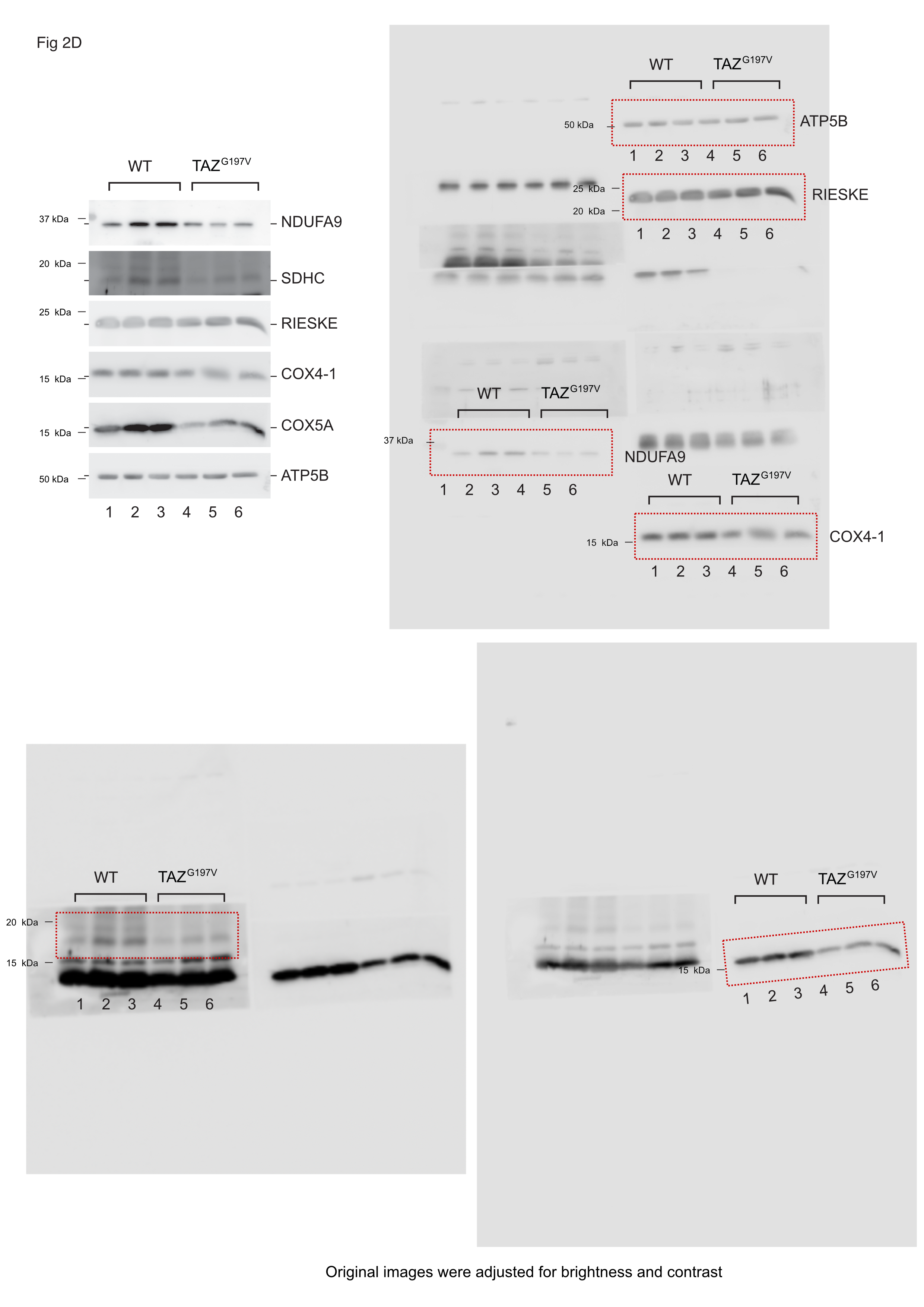

Supplement: Supplementary file 6 — Source Data for Figure 2 [file EMMM-15-e17399-s005.zip › Figure 2/2D/NDUFA9, SDHC, RIESKE, COX4-1, COX5A, ATP5B steady states .tiff]

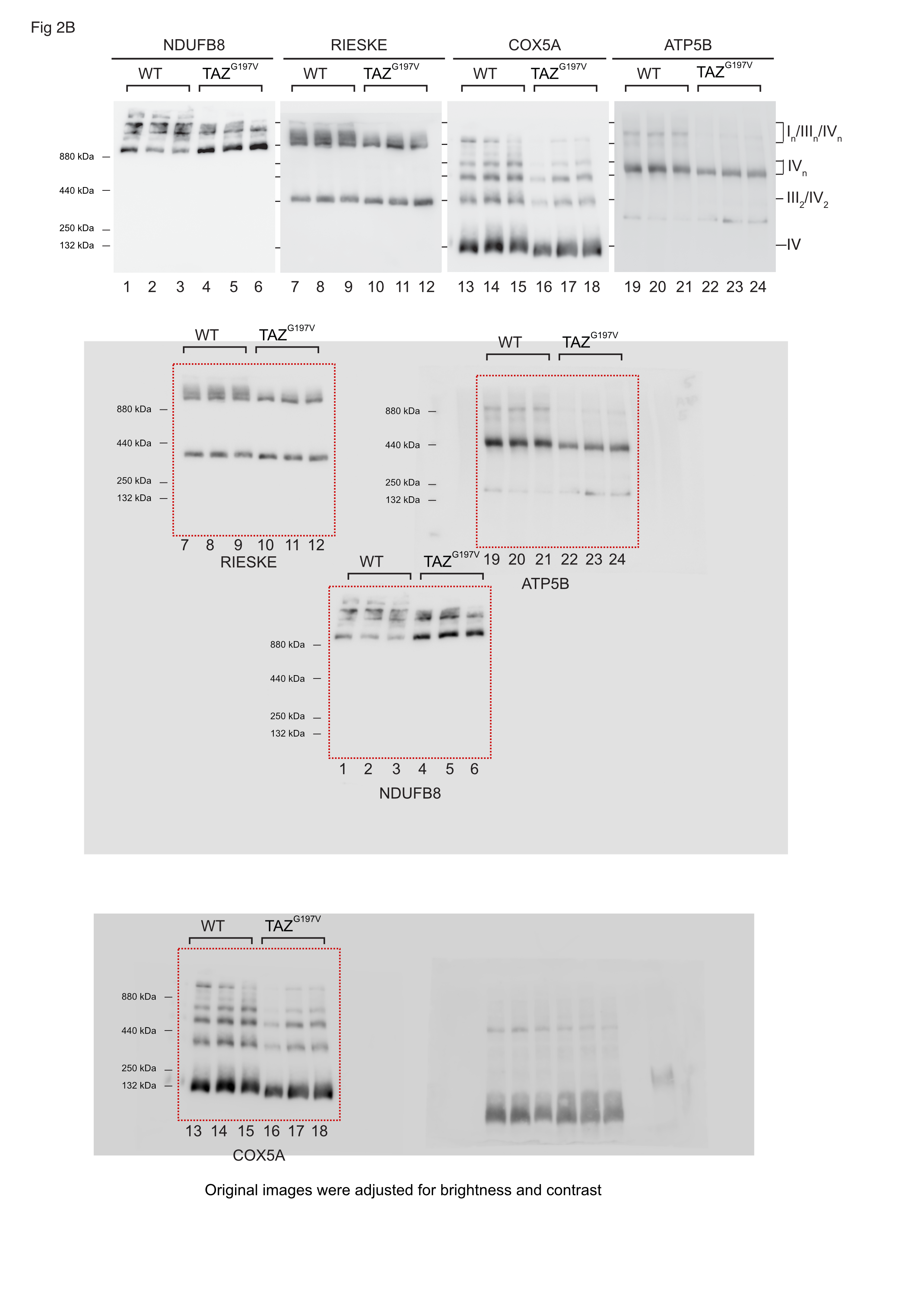

Supplement: Supplementary file 6 — Source Data for Figure 2 [file EMMM-15-e17399-s005.zip › Figure 2/2B/NDUFB8, RIESKE, COX5A, ATP5B - Blue Native Gels.tiff]

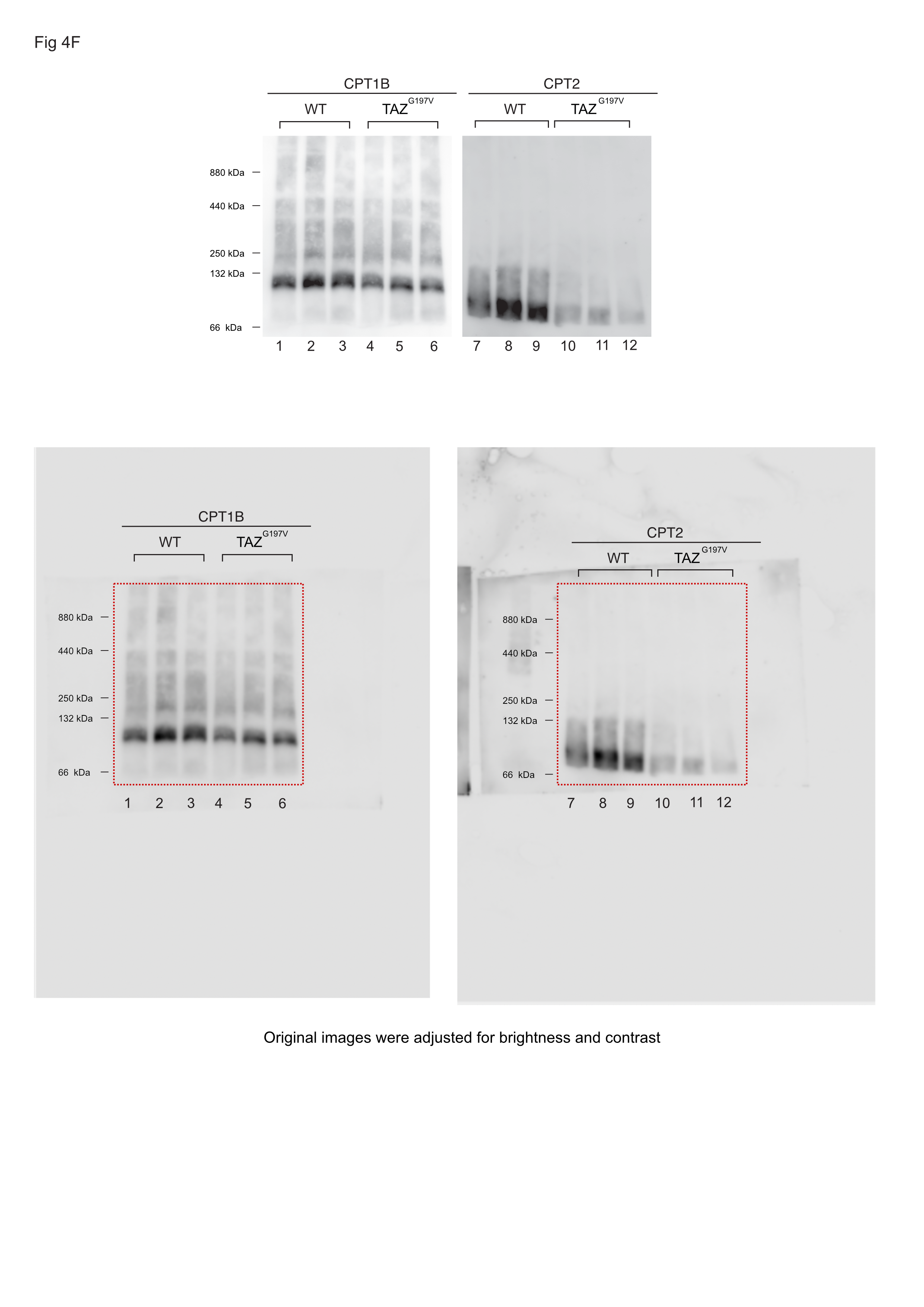

Supplement: Supplementary file 7 — Source Data for Figure 4 [file EMMM-15-e17399-s010.zip › Figure 4/4F/CPT1B, CPT2 Blue Native.tiff]

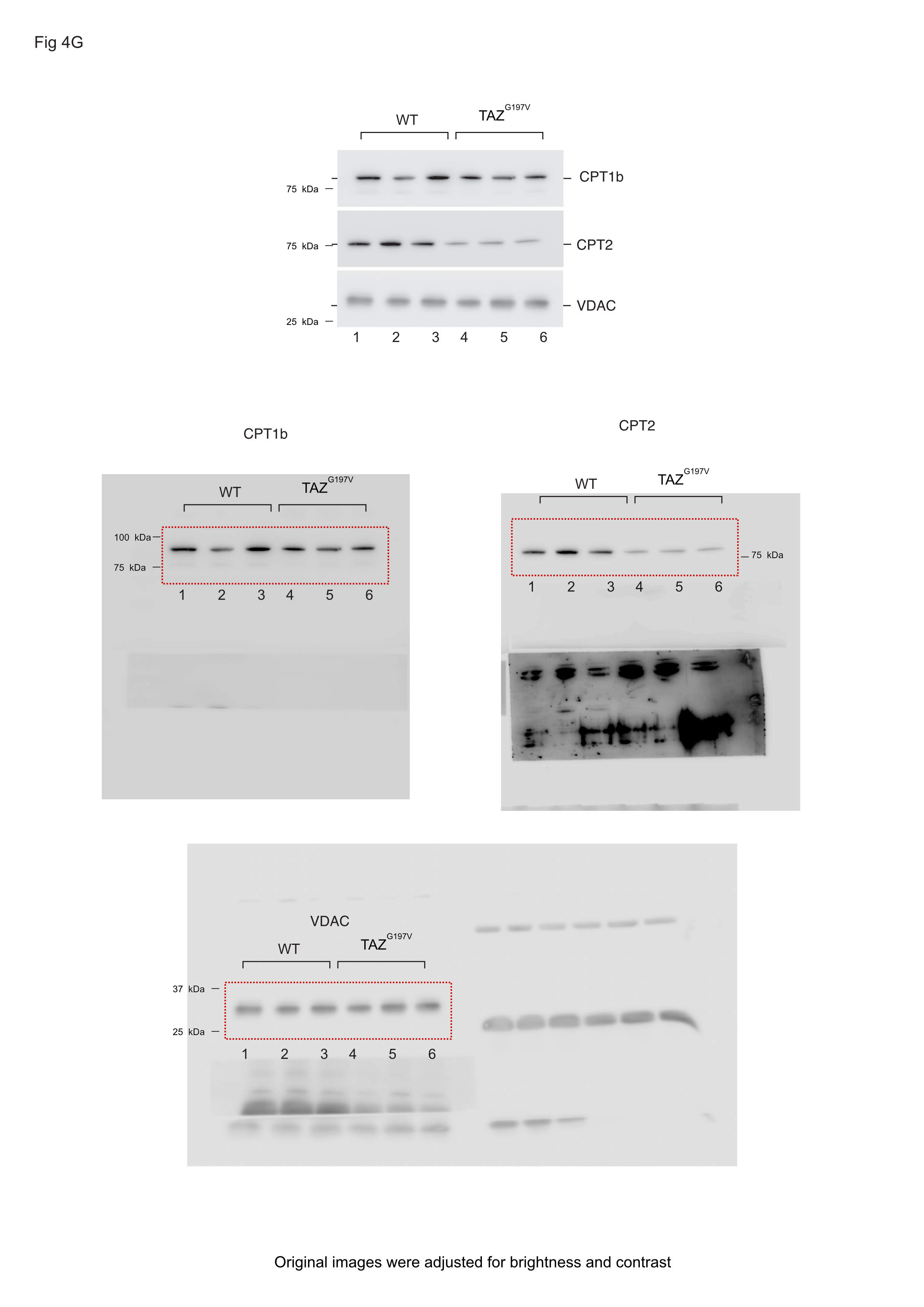

Supplement: Supplementary file 7 — Source Data for Figure 4 [file EMMM-15-e17399-s010.zip › Figure 4/4G/CPT1B, CPT2 Steady State .tiff]

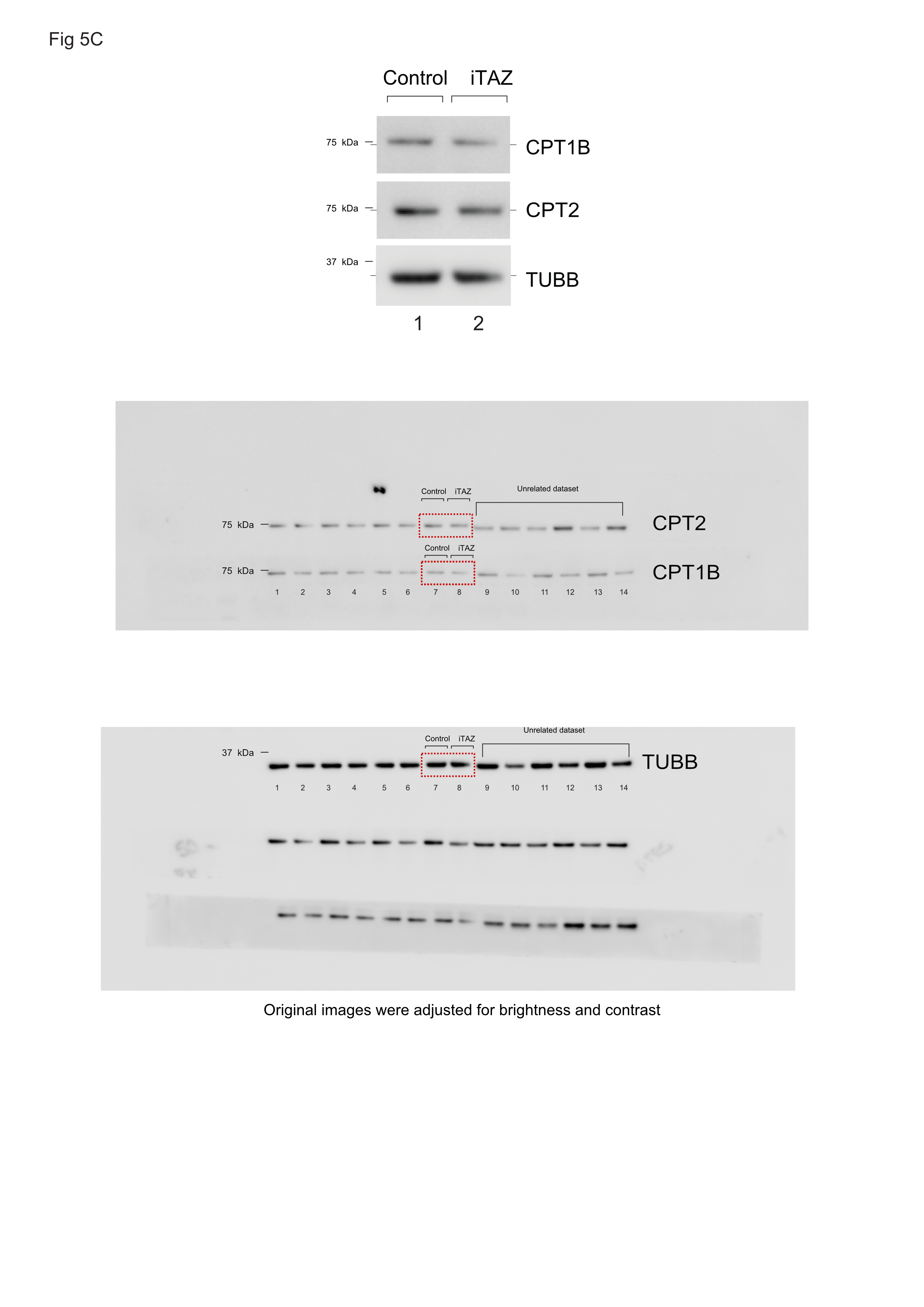

Supplement: Supplementary file 8 — Source Data for Figure 5 [file EMMM-15-e17399-s011.zip › Figure 5/5C/CPT1B, CPT2 Steady States iPSCS.tiff]

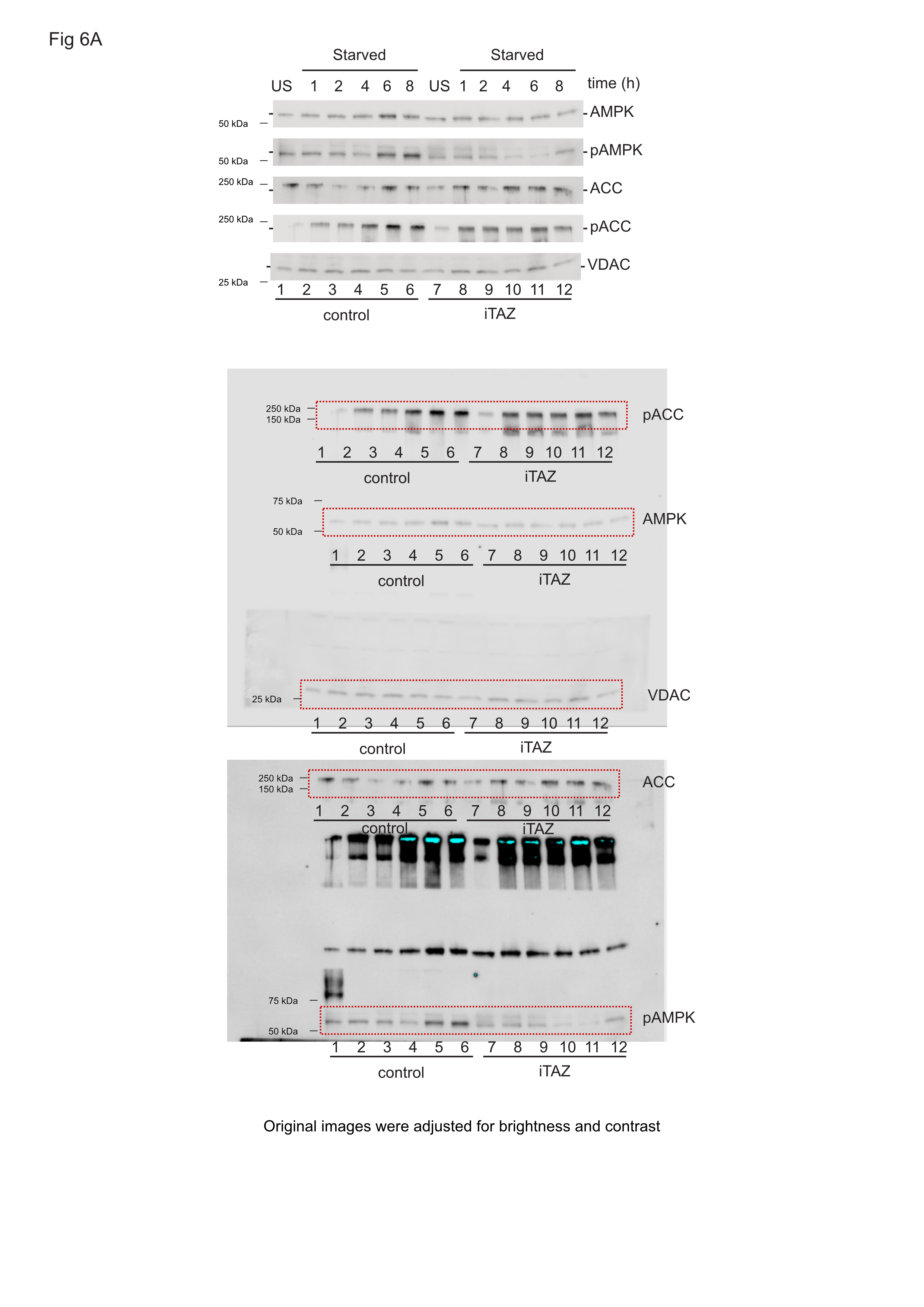

Supplement: Supplementary file 9 — Source Data for Figure 6 [file EMMM-15-e17399-s006.zip › Figure 6/6A/AMPK, pAMPK, ACC, pACC, VDAC Steady State.tiff]

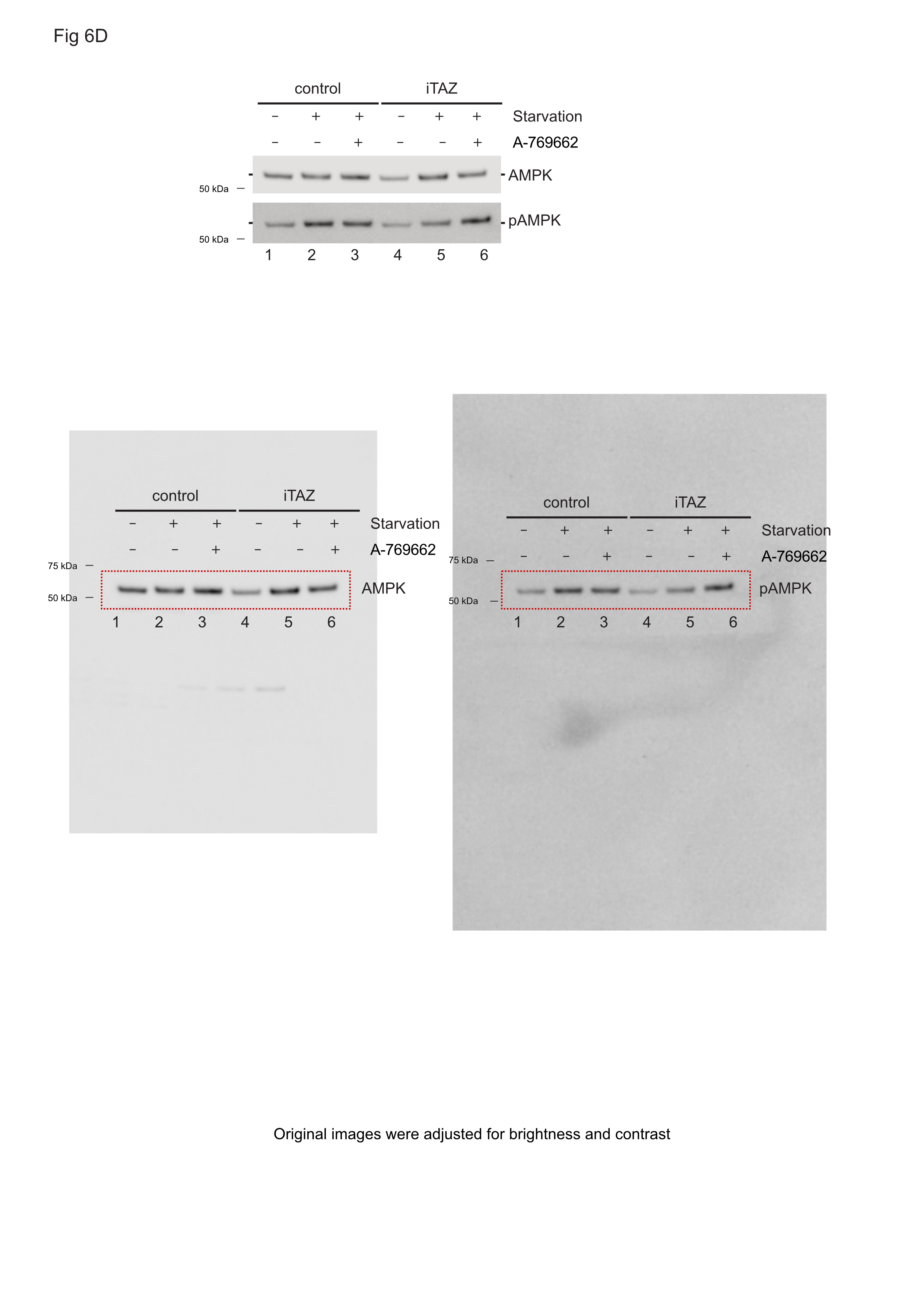

Supplement: Supplementary file 9 — Source Data for Figure 6 [file EMMM-15-e17399-s006.zip › Figure 6/6D/AMPK, pAMPK steady states with A-769662.tiff]
